# Computational Insights into PB1-F2 Mediated Modulation of VDAC1: An Allosteric Mechanism in Mitochondrial Dysfunction

**DOI:** 10.1021/acsomega.6c04195

**Published:** 2026-06-30

**Authors:** Sehrish Jamal, Syed Tarique Moin, Ulrich Kleinekathöfer

**Affiliations:** † School of Science, 84498 Constructor University, 28759 Bremen, Germany; ‡ Third World Center for Science and Technology, H.E.J. Research Institute of Chemistry International Center for Chemical and Biological Sciences, University of Karachi, Karachi 75270, Pakistan

## Abstract

The influenza-encoded virulence factor PB1-F2 has been implicated in a mitochondrial dysfunction of host immune cells through its interaction with the Voltage-Dependent Anion Channel 1 (VDAC1). This protein is a key outer mitochondrial membrane channel involved in ion and metabolite transport. Despite their well-known association, the precise interaction sites between these proteins remain unclear. In the current study, we employed multireplica all-atom molecular dynamics (MD), steered MD simulations, and docking approaches to investigate their mode of interaction. Our results reveal a putative binding site for PB1-F2 on the VDAC1 channel, as residue E73 has been reported to have physiological relevance in VDAC1 function; this region was initially explored as a potential interaction site. Furthermore, simulations based on multiple replicas revealed allosteric effects on VDAC1, increasing the dynamics of pore-lining residues and indicating a shift toward a probable closure-like conformation. We identify a stable binding interface at a global energy minimum, where PB1-F2 interacts via electrostatic interactions with the N-terminal pore-lining residues of VDAC1, which was also validated by voltage-driven simulations. We observed that due to the positive charge of PB1-F2, its interaction with the VDAC1 pore did not significantly affect the ionic conductance or selectivity of the channel. These findings provide molecular insights into how PB1-F2 modulates the function of VDAC1, reinforcing its role in modifying the permeability and possibly apoptosis induction. Understanding this interaction at an atomic level may inform novel strategies to deal with influenza-encoded virulence-induced dysfunctions of immune cells.

## Graphical abstract

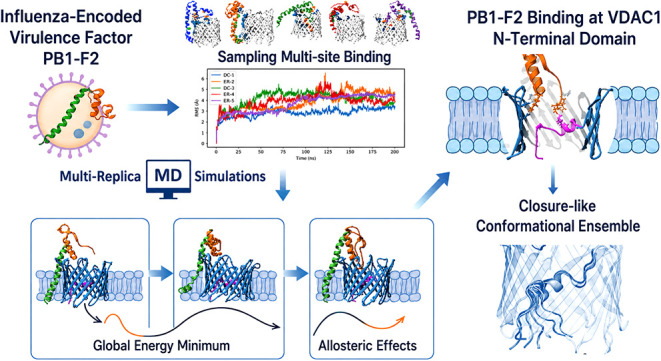

## Introduction

Respiratory viruses have emerged as a significant threat to human health in recent times, leading to unusually high rates of hospitalizations and mortality, and imposing a substantial economic and healthcare burden. Each year, seasonal influenza virus epidemics account for between 3 to 5 million severe cases and up to 6,50,000 deaths.^1^ The polymerase basic 1 frame 2 (PB1-F2) of Influenza A virus (IAV) stands out as an enigmatic and multifaceted virulence factor. Discovered in 2001, it was first identified as an “immune cell killer”, known for inducing apoptosis in host immune cells.^2,3^ PB1-F2 from all IAV strains comprises two conserved structural domains, an N-terminal domain (NTD) and a C-terminal domain (CTD), with an approximate total molecular weight of 11 kDa.^4^ Studies indicate that PB1-F2 exhibits a high degree of structural flexibility in aqueous solution but adopts an extended α-helical conformation in membrane-mimetic environments.^4^ The NTD contains two closely packed short α-helices, whereas the CTD forms a single extended α-helix. These domains are connected by a flexible, largely unstructured hinge region.^4^ Both the full-length protein and the isolated NTD are soluble at pH values ≤ 6, whereas the fragment containing CTD is soluble only under more acidic conditions (pH ≤ 3).^5^ The pathogenic effects of PB1-F2 are species and strain-specific, and have been extensively investigated.^6–11^ Studies have shown that PB1-F2 induces apoptosis by permeabilizing mitochondrial membranes, forming pores, and causing cytochrome c leakage through aggregation and oligomerization,^12^ preferentially affecting monocytes and macrophages. Its pro-apoptotic activity varies by viral strain, influenced by phosphorylation of residues T27 and S35, although specific residue roles remain unclear. Additionally, PB1-F2 interacts with mitochondrial proteins MAVS and TBK1, modulating RIG-I signaling pathways involved in antiviral responses.^13,14^ PB1-F2 also interacts with outer mitochondrial membrane protein, Voltage-dependent anion channel 1 (VDAC1), and inner mitochondrial membrane protein, adenine nucleotide translocase 3 (ANT3), and induces apoptosis during infection via activation of permeability transition.^3^ The detailed mode of action or the underlying mechanism for apoptosis induction via PB1-F2 in host immune cells is not well understood. However, since VDAC1 is localized in the outer mitochondrial membrane, PB1-F2/VDAC1 interaction might represent an important step in the initial stages of the PB1-F2-induced apoptotic activation.

Voltage-dependent anion channels (VDACs) are the prominent and abundant gated porins in the OMM (Outer Mitochondrial Membrane) that facilitate the transport of ions and metabolites.^15^ Due to their high ionic conductance, VDACs allow rapid equilibration of ions across the OMM, thereby preventing the establishment of a significant membrane potential.^16,17^ Malfunctions in VDACs that alter membrane permeability and ion flux have been associated with mitochondrial dysfunction and the induction of apoptosis.^18^ There are three VDAC isoforms, out of which VDAC1, a 283 amino acid protein of 31 kDa size, is the most abundant in humans.^19^ Structurally, the VDAC1 channel is a β-barrel composed of 19 β-strands and contains a largely unstructured N-terminal domain with two small helical segments, α1 and α2. The N-terminal domain is located within the barrel lumen and is stabilized through interactions with the barrel wall (β-strands 7–19). Previous reports have hypothesized that the N-terminal domain mediates channel closure through an unwinding mechanism^20^ or the movement of the α-helix toward the center of the channel pore,^21,22^ possibly with characteristic elliptic deformations of the barrel structure.^20–23^ In contrast, cross-linking experiments suggest that the helix is unlikely to completely dislocate from the pore.^24,25^ Nevertheless, the specific conformational changes associated with the closed state(s) remain elusive. Biochemical reports suggest that closed states of VDAC are voltage-driven, demonstrating lower anion permeability and no ATP permeability at higher membrane potentials. On the contrary, the dominant open state of VDAC is selective for anions and metabolites like ATP. The membrane permeability also depends on the VDAC1 binding partners, including those introduced into the cell by viral pathogens. Certain proteins and metabolites modulate the mitochondrial membrane potential by allowing the closure of VDAC1.^26–30^ These closed states are considered to be more permeable to calcium ions from the cytosol.^31^ Subsequently, this increase in calcium uptake aids in the ROS levels, which promote cell migration and invasion in lung cancers.^32^ Both VDAC1 and VDAC2 act as dynamic translocation platforms for the pro-apoptotic proteins Bak and Bax of the Bcl-2 family that facilitate the permeability of mitochondria toward cytochrome C, which could enhance or inhibit the apoptotic cascade. However, recent studies have predominantly highlighted VDAC2 as the principal isoform involved in this process.^33–39^

Considering the pro-apoptotic properties of PB1-F2, its potential interaction with mitochondrial proteins such as VDAC1 demands detailed investigation. However, despite growing insights into the structural features of PB1-F2, its precise molecular mechanism of action remains poorly understood due to the lack of an experimentally resolved structure. To date, only one study has reported a truncated, monomeric PB1-F2 acting as a nonselective ion channel.^40^ To address this gap, we previously modeled full-length PB1-F2 in its oligomeric forms and examined their potential role in membrane permeabilization and apoptosis induction^41^ using molecular dynamics simulations. In continuation, the current study explores an alternative apoptotic mechanism by focusing on the interaction of PB1-F2 with VDAC1. Employing structural data from known VDAC1-binding proteins, we modeled and analyzed PB1F2-VDAC1 complexes using docking and molecular dynamics simulations. These findings provide mechanistic insights into the PB1-F2/VDAC1 association and suggest a novel pathway through which PB1-F2 may modulate mitochondrial function and contribute to apoptosis.

## Methods

### System Setups and Simulation Details

The atomic coordinates of PB1-F2 from the H1N1-IAV strain in its monomeric state were extracted from previously proposed oligomeric forms.^41^ The accession ID of the isolated sequence is QCT08405 (A/Texas/9437/2019), sourced from the Influenza Virus Database of NCBI.^42^ For the homology model of human VDAC1 (Uniprot ID: P21796), we used the mouse VDAC1 (PDBID: 3EMN)^43^ as a template, employing MODELER 10.5 ^44^ for the construction.

The modeled structures were separately embedded in a mixed bilayer system composed of Palmitoyl-oleoylphosphatidylcholine (POPC) and Palmitoyl-oleoylphosphatidylethanolamine (POPE) lipids in a 2:1 ratio, utilizing the CHARMM GUI server.^45^ This membrane composition was selected as a commonly used model system that balances computational tractability with biophysical relevance and enables comparison with previous VDAC simulation studies.^46–49^ We note, however, that the native mitochondrial outer membrane is compositionally more complex, containing additional lipid species such as cardiolipin and phosphatidylinositol and exhibiting leaflet asymmetry.^50^ Accordingly, the present membrane model represents a simplified system, and potential lipid-specific effects on the PB1-F2/VDAC1 interaction cannot be ruled out.

PB1-F2 was oriented perpendicular to the membrane plane (along the bilayer normal, *z*-axis) and anchored via the Membrane Targeting Sequence (MTS) spanning residues 55–90. Similarly, VDAC1 was oriented using the OPM server^51^ to ensure proper alignment within the membrane environment, while keeping the important E73 residue in its deprotonated state. The lipid-packed PB1-F2 and VDAC1 systems were then solvated using the TIP3P water model,^52^ with the addition of 0.15 M KCl and some additional ions to reach charge neutrality of the systems.

The steepest-decent algorithm was utilized to remove steric clashes within the systems via 5000 energy-minimizing steps. Afterward, equilibration was carried out in six stages. The first two steps comprised NVT equilibrations in a total of 250 ps at a 1 fs time step. Where the positional restraints of 4000 kJ mol^–1^ nm^–2^ force constant were applied to protein backbone atoms and 2000 kJ mol^–1^ nm^–2^ to protein side chains, which subsequently relaxed to 2000 kJ mol^–1^ nm^–2^ and 1000 kJ mol^–1^ nm^–2^ in the later stage. Likewise, the phosphorus atoms of the phospholipid were subject to positional restraints of 1000 kJ mol ^–1^ nm^–2^, relaxing to 400 kJ mol^–1^ nm^–2^ force constant, whereas dihedral restraints of 1000 to 400 kJ mol^–1^ nm^–2^ were applied. Moreover, the systems were exposed to a controlled temperature of 303.15 K, using the Berendsen weak coupling method.^53^ The remaining four equilibration stages were then carried out using an isothermal–isobaric NPT ensemble with a total simulation time of 375 ps. The first 125 ps of the simulation time were conducted using a time step of 1 fs, followed by the rest of the three stages for 250 ps using a 2 fs time step. The positional restraints on the protein backbone atoms were gradually relaxed from 1000 to 50 kJ mol^–1^ nm^–2^, similarly for the side chain atoms, from 500 to 0 kJ mol^–1^ nm^–2^, for the phosphorus atoms from 400 to 0 kJ mol^–1^ nm^–2^, and the dihedral restraints were relaxed from 200 to 0 kJ mol^–1^ nm^–2^. The Berendsen weak-coupling barostat^53^ was employed to maintain a pressure of 1 bar. After passing through the equilibration stages, the simulations in the production stage were performed without restraints. During the production runs, a Nosé-Hoover thermostat with a coupling time constant of 1.0 ps and a Parrinello–Rahman barostat with a coupling time constant of 5 ps and a value of 4.5 × 10^–5^ bar^–1^ for the compressibility κ_p_ were employed to maintain a temperature of 303.15 K and a pressure of 1 bar. The lengths of hydrogen bonds were constrained using the LINCS algorithm.^54^ For nonbonded interactions, a cutoff of 12 Å was applied, with neighbor lists handled using the Verlet cutoff scheme. The long-range electrostatics was treated using the Electrostatic Particle Mesh Ewald (PME) approach.^55^ Furthermore, periodic boundary conditions were implemented in all directions. A time step of 2 fs was applied during the production runs. Both the PB1-F2 and the VDAC1 systems were first simulated for 200 ns, followed by two independent production runs of 2 μs each. All MD simulations were performed using GROMACS version 2022.3,^56,57^ employing the CHARMM36m force field.^58^ CHARMM36m force field was selected because it was specifically refined to improve backbone conformational sampling for both folded proteins and intrinsically disordered proteins,^58,59^ making it suitable for the mixed VDAC1 (folded) and PB1-F2 (partially disordered) system studied here. We note that some studies report residual biases for certain IDPs, for instance, increased compactness or helicity;^60,61^ therefore, interpretations of flexible regions were made cautiously and based primarily on persistent interfacial interactions rather than isolated secondary-structure fluctuations.

### Steered Molecular Dynamics

Taking the significance of the E73 amino acid residue of VDAC1 in receptor binding into account,^62^ PB1-F2 was placed in a membrane environment at a distance of about 38 Å from VDAC1 facing E73. Again, the membrane had a lipid composition of POPC: POPE (2:1). To equilibrate this system, the same MD protocol was performed and followed by a 200 ns long production run. Subsequently, a steered MD run was performed by pulling the CTD of PB1-F2 toward VDAC1 porin within the *x*–*y* plane. The difference of the centers of mass of the CTD of PB1-F2 and the Cα atoms of VDAC1 was used as a reaction coordinate. The pulling was conducted at a rate of 1Å/ns using a force constant of 2.39 kcal mol^–1^ Å^–2^. From the SMD trajectory, a representative snapshot corresponding to the point of strongest interaction (maximum force) was extracted and subjected to a 2 μs-long unbiased MD simulation following the previously adopted simulation protocol. A second steered MD run was set up by taking a snapshot from an unbiased run, and pulling was performed on the N-terminus of PB1-F2 along the membrane normal (*z*-axis), toward the intermembrane space (IMS) side of VDAC1. Therefore, the reaction coordinate was defined between the N-terminus of PB1-F2 and the center of mass of Cα atoms of VDAC1, at a rate of 1Å/ns with a force constant of 2.39 kcal mol^–1^ Å^–2^. Subsequently, five snapshots were collected during the pulling simulation at successive intervals as the starting points for unbiased simulations. Each of these simulations was performed for 200 ns to capture the dynamics of VDAC1 in response to the presence of PB1-F2.

### PB1-F2-VDAC1 Docking

Protein–protein docking was adopted as the second strategy to model the PB1-F2 and VDAC1 complex. The ClusPro 2.0 server^63–66^ was employed to generate the docking conformations via three steps. In the first step, the server aims for rigid body docking by sampling millions of conformations. In the second step, conformations are clustered based on the RMSD from the 1000 lowest energy complexes, representing the largest clusters for the most probable protein–protein complexes. Subsequently, the complexes are refined using energy minimizations.

ClusPro employs PIPER,^67^ a Fast Fourier Transform (FFT) based docking framework that incorporates a pairwise interaction potential as the scoring function. It generates conformations based on four distinct models: (1) balanced, (2) electrostatic-favored, (3) hydrophobic-favored, and (4) van der Waals + electrostatics. The balanced model is typically ideal for enzyme–inhibitor complexes, whereas the van der Waals + electrostatics model is best suited for proteins that differ significantly from the typical protein–protein complexes used in the parametrization of the other three models. Given that PB1-F2 and VDAC1 are relatively underexplored binding partners, the top five protein–protein complexes generated using the van der Waals + electrostatics model were further refined based on the criterion that the PB1-F2 membrane-targeting sequence (MTS) should remain in the membrane vicinity. These top 5 docking conformations (DC1–5) were further subjected to 200 ns unbiased MD simulation runs, followed by trajectory analysis. Based on a thorough analysis, Docking Conformation 1 (DC1) was selected for more conformational space exploration for a longer simulation time of 10 μs, divided into 10 replicates. The unbiased MD simulation protocol remained consistent throughout the study.

### Network Analysis

In the dynamical network analysis, each residue of the protein complex was represented as a node, defined by the position of its Cα atom. Pairwise distances between nodes were computed throughout the trajectory, and two nodes were considered to be in contact if the minimum distance between them was less than 4.5 Å for at least 75% of the simulation time. This contact-based network was used to identify structural communities, which are clusters of residues that interact closely and function cooperatively. Changes in the network architecture influenced the identification of these communities and the corresponding suboptimal pathways. The network analyses were performed using the Dynamic Network Analysis Python package.^68^

### Principal Component Analysis (PCA Analysis)

To investigate the dominant motions of PB1-F2 and VDAC1 during the simulations, principal component analysis (PCA) was performed using the Bio3D package.^69^ For each system, Cα atoms were used to capture the overall backbone dynamics. The simulation trajectories were aligned to their respective reference structures to eliminate global translational and rotational motions. A covariance matrix of atomic positional fluctuations was constructed from the aligned Cartesian coordinates, and a PCA was applied to extract the major modes of motion. The analysis focused on the first three principal components, which captured the most significant collective fluctuations of the protein structures.

### Ellipticity of the VDAC1 β-Barrel

The ellipticity was quantified by comparing the major axis (a) and minor axis (b) of an ellipse fitted to the cross-sectional shape of the Cα-atoms of VDAC1 β-barrel structure, using the formula $\mathrm{ellipticity}=1-\frac{b}{a}$ . A perfectly circular β-barrel corresponds to an ellipticity value of 0, while increasing values indicate greater deviation toward an elliptical shape, approaching 1.

### Applied Field MD Simulations

To analyze the ion conduction properties of VDAC1, applied-field MD simulations were conducted by following standard procedures.^70,71^ External voltages of ± 100 mV, ± 75 mV, ± 50 mV, and ± 25 mV were applied, which can be applied in electrophysiology experiments and mimic potential differences between the cytosolic and the IMS sides of the VDAC channel. The effect of applied fields on the joint PB1-F2-VDAC1 complexes was also evaluated. To this end, one of the metastable states (MS1) derived from the extended unbiased simulations of DC1 was taken and subjected to external voltages of ± 100 mV and ± 75 mV. The resulting trajectories were subsequently analyzed concerning ion conduction, structural, and dynamical properties. A quick overview of all MD Simulations carried out in this study is presented in Table 1.

**Table 1:** Summary of MD Simulations Conducted in the Current Study

	parameters	PB1-F2	VDAC1		SMD		DC1	
system preparation	no. of atoms	131,146	152,069		317,282		180,842	
	no. of lipids	upper leaflet: 183	upper leaflet: 252		upper leaflet: 252		upper leaflet: 249	
		lower leaflet: 183	lower leaflet: 252		lower leaflet: 252		lower leaflet: 249	
	system size along the *x*-*y* axis	110 Å	135 Å		135 Å		135 Å	
molecular dynamics (MD) simulations	simulation Time	2.2 μs	unbiased	2.2 μs	unbiased	3 μs	unbiased	10.2 μs
			at applied voltage	200 ns × 8			at applied voltage	200 ns × 4
				= 3.8 μs				= 11 μs

## Results and Discussion

### PB1-F2 and VDAC1: Distinct Molecular Entities

PB1-F2 is a structurally flexible protein whose conformation depends on strain and environment.^4^ In membrane-mimetic systems, the mitochondrial targeting sequence (MTS), i.e., residues ∼55–85 located in CTD, adopts a stable α-helical structure, while the NTD retains partial flexibility.^4^ As no experimentally resolved monomeric structure is currently available, the PB1-F2 model used here was obtained by extracting a protomer from a previously characterized oligomeric membrane-associated structure^41^ and subsequently equilibrating it before investigating its interaction with VDAC1.

To validate the structural stability of this protomer, we first evaluated its independent dynamical behavior in the membrane environment through simulations with a total length of 2.2 μs. The prominent dynamical features of PB1-F2 in the lipid environment were analyzed by identifying the most collective and correlated atomic motions, as captured by the principal components. These motions were then projected along the eigenvectors with respect to the largest variance in the conformational space (Figure S1).^69^ The conformational shifts associated with these eigenvectors involve a high mobility in the NTD of the PB1-F2 structure (Figure 1A–C). PC1 dictates a sideways displacement of the N-terminal end in the membrane plane, while PC2 captures its motion in the direction of the surface normal. In contrast, PC3 differs by encompassing a movement around the G24 residue. The membrane-embedded CTD, interestingly, remains stable. We also evaluated the tilt angle of the CTD with respect to the membrane normal, which showed angular fluctuations in the range from 25° to 40° (Figure 1D). Similarly, the tilt angle between S34, D50, and the membrane plane showed a transition from an open conformation to a closed conformation by switching from 40° to 140°. This closed conformation was further confirmed via angle calculation between S34, D50, and P62, which was around 40° (Figure 1E,F). The specific closed conformation (Figure 1G) was observed in the first 200 ns equilibration phase. Through this analysis of the dynamical behavior of PB1-F2, we characterized features consistent with its intrinsically disordered nature in the monomeric form, highlighting its adaptive response to the membrane environment. Since the CTD of PB1-F2 is already known for its membrane targeting capabilities,^72^ the observed dominant motions in the NTD further underscore its functional significance. This dynamic flexibility could be crucial for its role in potential interactions with other mitochondrial proteins, such as VDAC1.

**Figure 1:**
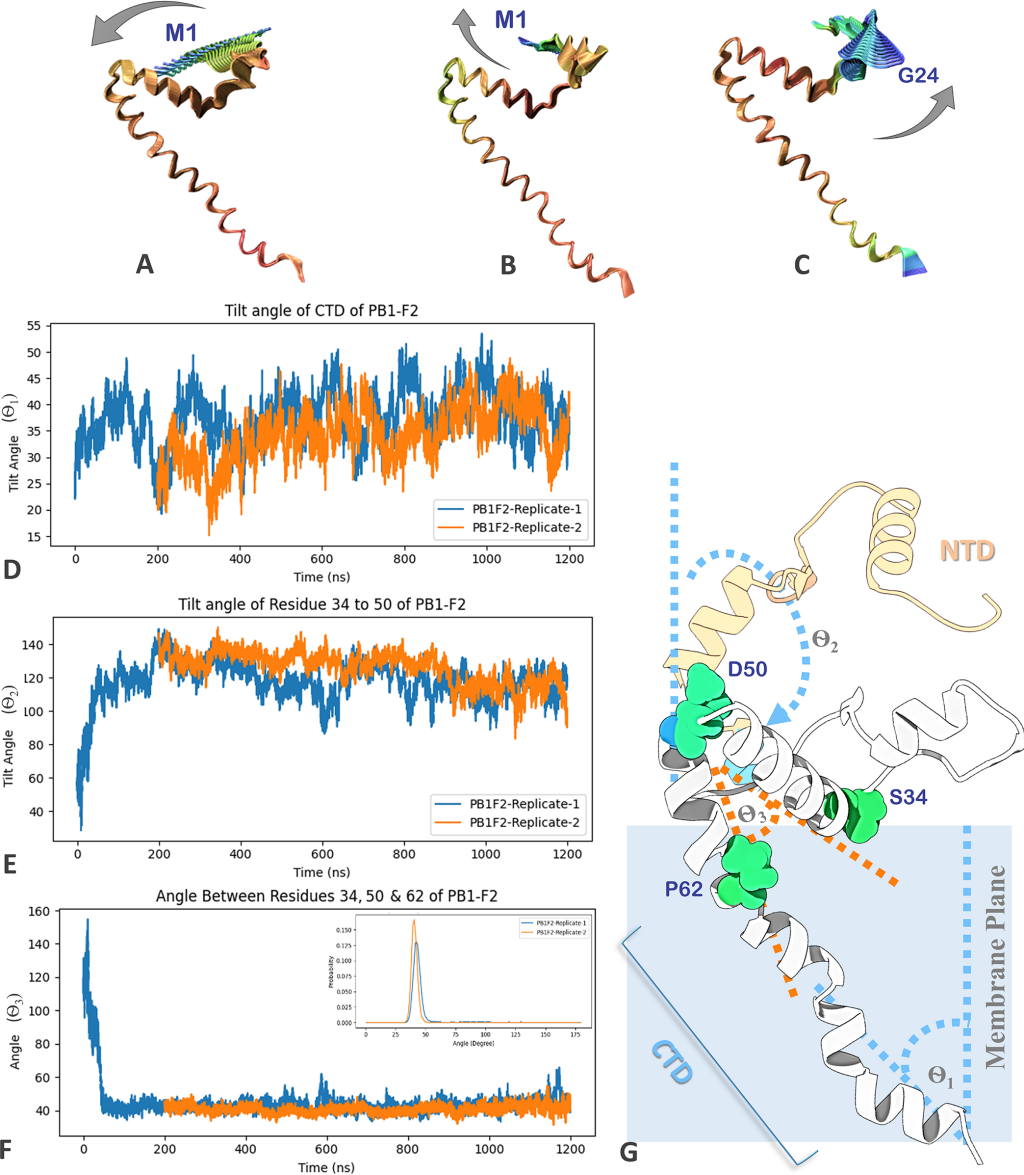
(A) Principal Component Analysis of PB1-F2 depicting the highly movable amino acids along PC1, (B) PC2, and (C) PC3. (D) Tilt angle of the CTD of PB1-F2 with respect to the membrane normal. (E) Tilt angle between amino acid residues 34, 50, and the membrane normal. (F) Angle between amino acid residues 34, 50, and 64, along with (G) a visual depiction of PB1-F2 in cartoon representation, highlighting the analyzed quantities.

Moreover, we also simulated VDAC1 and performed a PCA on its respective 2.2 μs simulation trajectories. The overall charge on this porin is +3 elementary charges *e*, as illustrated in Figure 2B, with the negatively charged amino acid E73 protruding toward the membrane vicinity, providing an attachment site for hexokinases^73^ and cholesterol.^62,74^ The electrostatic field lines in Figure 2B yield insights into the electrostatic potential of VDAC1, which can influence the nature of its interacting partners. Whereas, the high mobility points in the VDAC1 structure (Figure 2C–E) were mainly comprised of loop regions, which include amino acid residues E36, A134, A160, K161, N214, V268, and N269, present on the β-strands 1, 9, 11, 15, and 19. VDAC1 has 19 antiparallel β-sheets forming a hydrophilic pore with an α-helical N-terminal domain essential for gating.^75^

**Figure 2:**
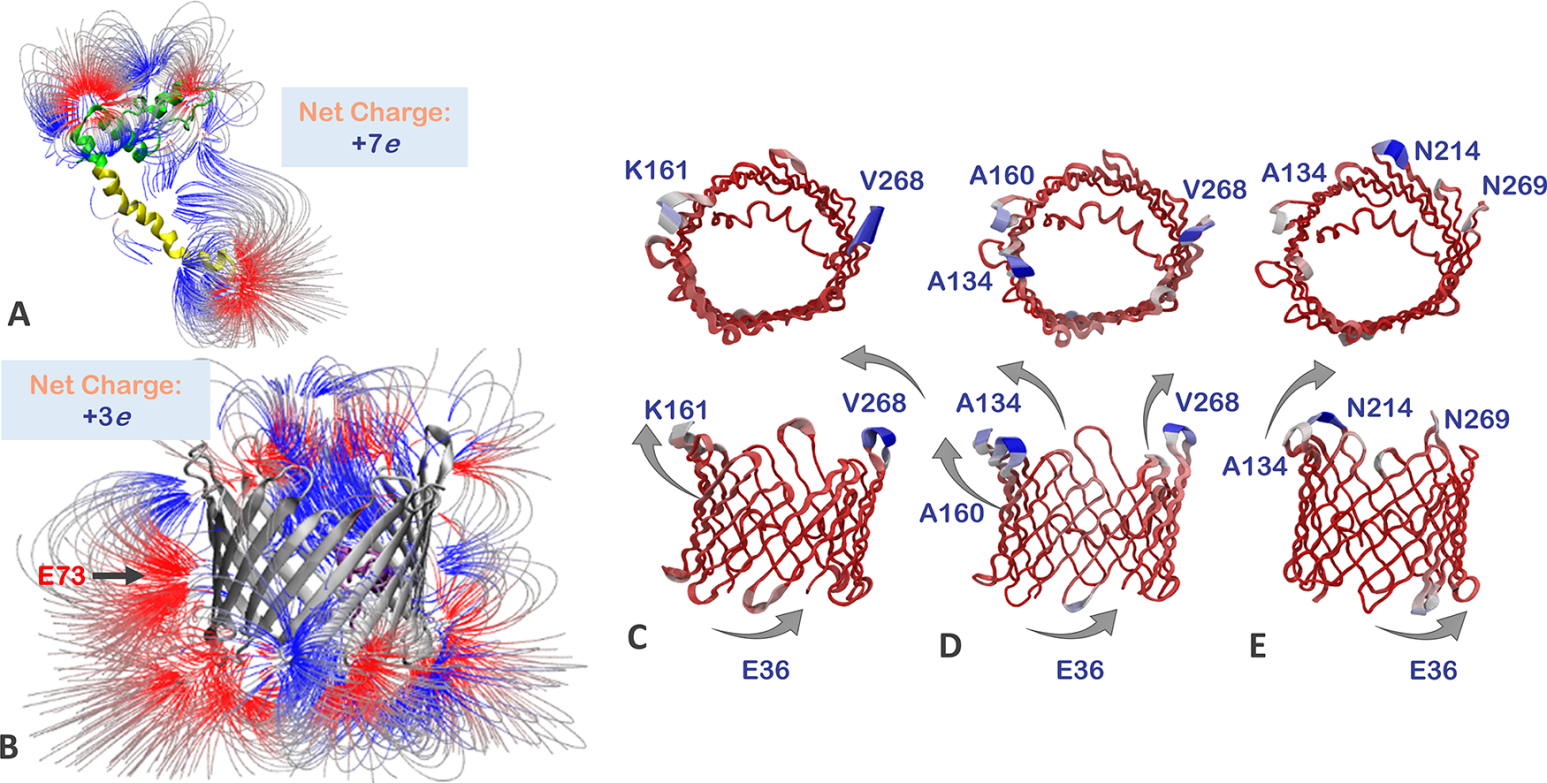
(A) Electrostatic field lines around PB1-F2, where blue lines represent fields generated by positively charged amino acid residues and red lines by negatively charged amino acid residues, along with the depiction of the CTD in yellow and the NTD in green. (B) Electrostatic field lines around VDAC1, along with the biologically important negatively charged E73 amino acid residue protruding toward the membrane region. Moreover, (C) a Principal Component Analysis of VDAC1 is shown for PC1, (D) PC2, and (E) PC3.

Since the pore lumen favors interactions with electronegative moieties, electropositive proteins like PB1-F2 may experience some electrostatic repulsion when approaching the N-terminal domain to block the pore, in order to mimic the negatively charged proteins like tubulin or α-synuclein, which are known to directly interact with and transiently occlude the VDAC pore.^28–30^ However, in such cases, potential allosteric effects cannot be excluded, as protein binding at one region of VDAC may induce conformational changes that influence its functional state.

### Mapping the Binding Interfaces of PB1-F2 and VDAC1

Next, we investigated the interaction between PB1-F2 and VDAC1. To assess structural robustness, we first performed an AlphaFold3-based prediction^76^ of the PB1-F2/VDAC1 complex. While the overall fold confidence was moderate (pTM = 0.71), the predicted interface confidence was low (ipTM = 0.41), indicating limited reliability of the predicted protein–protein interaction. This likely reflects the intrinsic flexibility and membrane-associated nature of PB1-F2.^4,41^ Therefore, the interaction models proposed in this study are primarily based on physics-based approaches.

To explore possible binding modes, we first performed SMD simulations by applying a force constant of 2.39 kcal mol^–1^ Å^–2^ to pull PB1-F2 toward VDAC1 (SMD1). The pulling was applied to the membrane-targeting sequence (MTS) located in the CTD of PB1-F2, guiding the protein laterally toward the VDAC1 barrel in a direction parallel to the membrane plane. Residue E73 served as a structural reference within the barrel wall. The glutamate residue at position 73 (E73) has consistently been highlighted as a critical residue in channel regulation.^62,77–79^ Located on the membrane-facing side of the VDAC1 barrel, E73 has been reported to promote local membrane thinning,^23,50^ which may facilitate the insertion of α-helical segments into the membrane, in addition to contributing to other physiological processes.^78^ E73 has also been identified as a key residue involved in hexokinase binding.^73,80^ The PB1-F2/VDAC1 complex obtained from the SMD run was simulated for an additional 2 μs. The analysis suggests that the short-range interactions are dynamic, with an average interaction energy of approximately −6.15 kcal/mol (Figure S2A). The hydrogen bond autocorrelation function exhibits a rapid decay, highlighting the transient nature of individual hydrogen bonds. However, the calculated forward (formation) rate of 0.1/ns, obtained from fitting the decay of *C*(*t*), indicates that while these bonds are short-lived, new hydrogen bonds form relatively infrequently during the simulation (Figure S2–B). Further analysis of the hydrogen bond numbers confirmed their intermittent nature, with fluctuations observed throughout the simulation. At the same time, approximately three hydrogen bonds remained relatively stable over time (Figure S2–C). This suggests that PB1-F2 interacts with VDAC1 primarily through transient hydrogen bonding rather than forming stable interactions when positioned to face E73.

To gain deeper insights, residual contacts between PB1-F2 and VDAC1 were analyzed to identify the residues involved in potential hydrogen bonding or electrostatic interactions. The analysis revealed that specific residues of PB1-F2, such as *R*32, P33, S34, and L38, interact with specific VDAC1 residues, including N79, P105, and N106 (Figure 3A,B), while maintaining the PB1-F2 in a compact or folded conformation. Interactions are dominated by residues on β5 and the loop of β7 of VDAC1. Interestingly, the residue E73 on VDAC1 does not contribute significantly to any direct contact with PB1-F2. This prompted us to better understand the dynamics of E73, so we evaluated the radial distribution function (RDF) with respect to water molecules, lipid head groups, and PB1-F2 around E73. The RDF analysis revealed spatial proximity between PB1-F2 and E73 (∼0.5 nm) (Figure 3C); however, the absence of direct interactions suggests that PB1-F2 may exert its effects indirectly by modulating the local hydration or lipid arrangement around E73. Interestingly, the hydration peak of water molecules near E73 (∼0.3 nm) indicates that this residue remains well-solvated. This solvation is potentially facilitated by the positively charged PB1-F2, which could help stabilize the water shell around E73 (Figure 3D).

**Figure 3:**
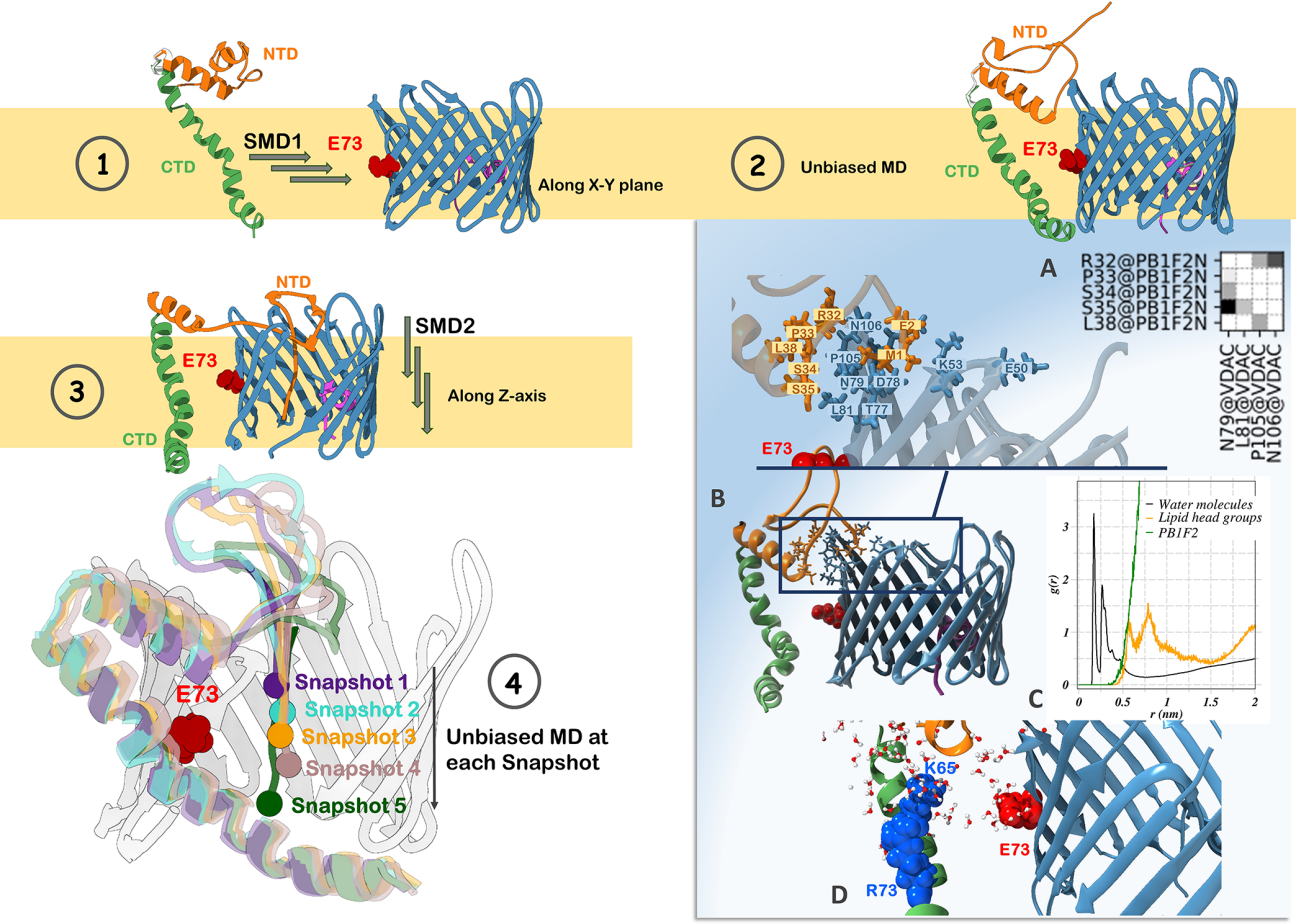
(1) Steered MD along the *x*–*y* plane, pulling CTD of PB1-F2 toward E73 of VDAC1, (2) followed by 2 μs unbiased MD. (3) Steered MD along the *z*-axis, pulling NTD of PB1-F2 toward the IMS side of VDAC1. (4)­Five snapshots were captured during the second pulling simulation for subsequent independent 200 ns unbiased MD runs. (A) The right panel shows the contact map of residual contacts between PB1-F2 and VDAC1, with (B) a magnified cartoon depiction of these contacts over the course of a 2 μs unbiased simulation after SMD1. (C) Radial distribution function of important E73 residue with respect to water molecules, lipid head groups, and PB1-F2 in the simulation system, followed by (D) its visual depiction. PB1-F2 and VDAC1 are depicted in cartoon representations with PB1-F2 CTD colored in green and the NTD in orange. β-barrel structure of VDAC1 is colored in blue, whereas the N-terminal helix is colored in purple. The important E73 residue of VDAC1 is depicted by a red sphere, and the positive residues K65 and R73 of PB1-F2 by blue spheres.

Notably, in the simulation of the PB1-F2/VDAC1 complex, the VDAC1 channel lumen and its N-terminal α-helical region remained largely unperturbed. We considered the possibility that the NTD of PB1-F2 probably inserts into the lumen and perturbs the electrostatics and transport properties of the channel. We therefore conducted a second steered molecular dynamics simulation pulling the NTD through the VDAC1 pore (SMD2). Snapshots randomly captured during SMD2 were further subjected to five 200 ns unbiased MD simulations to assess the stability of the interaction interface at each instance. A complete schematic approach for both steered MD runs is illustrated in Figure 3. A detailed analysis of the SMD2 simulations, including the stability of NTD interactions, is provided in the Supporting Information (Figures S3,S4). The results indicate that the NTD does not form a stable lumen-bound state under the simulated conditions, although transient contacts are observed in some snapshots.

While the SMD-based approach provides a possible binding mode of PB1-F2 and VDAC1, to uncover other possible binding interfaces, docking was also used to identify the specific residues in each protein that contribute to their interaction, providing insights into the key binding interfaces, such as electrostatic or hydrophobic interactions. By generating and ranking multiple docking conformations based on binding energies and cluster size, we determined the most likely physiologically relevant complexes. The best five docked conformations were selected, and the observed binding interfaces examined (Table 2). We further validated them through MD simulations. The binding interfaces were analyzed together with their key interaction hotspots, providing attachment sites in both the CTD and the NTD of PB1-F2. The interface RMSD of all the docked conformations is presented in Figure 4A,B, showing an increase in the interfacial area, except DC1, i.e., a substantial stabilization.

**Figure 4:**
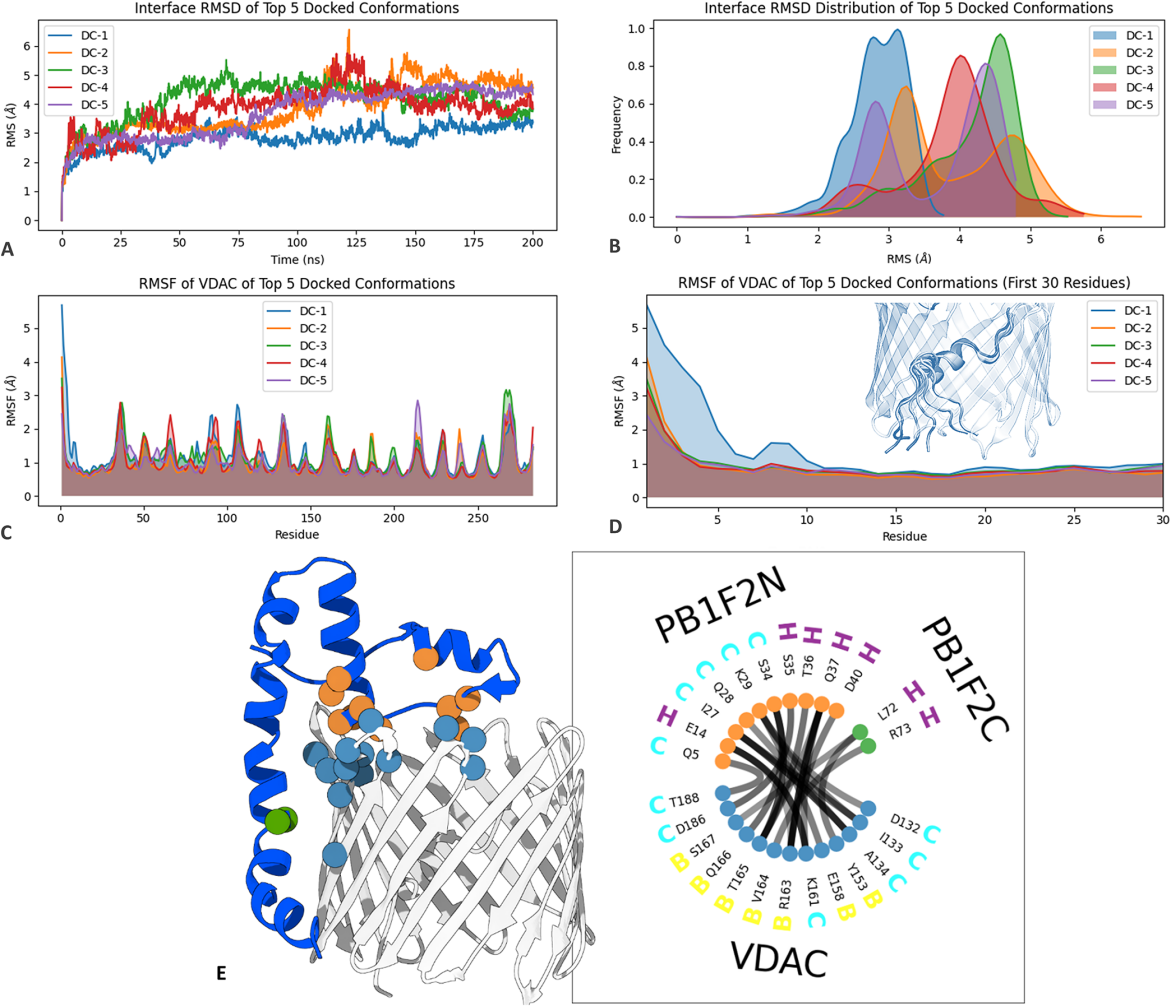
(A) Interface RMSD of the top five docked conformations between PB1-F2 and VDAC1 along 200 ns-long MD runs, along with (B) their frequency. (C) Residual RMSF of VDAC1 in the top five docking conformations, with (D) a magnified view of residual fluctuations of the N-terminal α-helical domain of VDAC1. (E) Cartoon representation of the PB1-F2/VDAC1 complex from DC1, along with a wheel representation of contacting residues with their secondary structure (H: helix, C: coil, and B: β sheet), with only contacting residues represented as spheres (color-coded based on protein domains).

**Table 2:** Selected Docked Conformations (DC), Showing Cluster Size, Representative Structures, with Their Weighted Docking Score, Interface Area, Number of Hydrogen Bonds and Salt Bridges at the Interface, and the Interacting Residues from PB1-F2 and VDAC1

cluster/docked conformation (DC)	members	representative	weighted score (kJ/mol)	interface area (Å^2^)	no. of hydrogen bonds at interface	no. of salt bridges at interface	residues of PB1-F2 at the interface	residues of VDAC1 at the interface
**1**	48	center	–157.7	728.1	8	7	Q5, E14, I27, Q28, K29, S34, S35, T36, Q37, D40, L72, R73	D132, I133, A134, Y153, E158, K161, R163, V164, T165, Q166, S167, D186, T188
		lowest energy	–157.7					
**2**	32	center	–141.9	904.0	2	2	G24, Q26, R59, S63, N66, L77, W80, K81, N84, W88	W149, Y153, V164, T165, Q166, S167, F169, Y173, E177, F178, V184, Q196, K197, V198, G213, S215
		lowest energy	–161.5					
**3**	21	center	–164.6	1289.5	13	6	G22, S23, Q25, Q26, I27, K29, L30, S34, Q48, D50, N66, Q69, L72, R73	A8, T6, F18, R15, T107, K109, Q154, R163, V164, N183, N185, D186, G187, E189, N207, N216, R218
		lowest energy	–164.6					
**4**	16	center	–150.4	807.1	14	4	L30, G31, S34, T36, Q37, W61, P62, K65, N66, Q69, L72, R73	T188, W210, T211, A212, G213, N214, S215, T217, R218, S240
		lowest energy	–150.4					
**5**	15	center	–157.2	1291.2	14	4	Q5, Q19, R20, S23, Q26, I27, S34, S35, T36, D40, P62, K65, N66, Q69	R15, F18, T19, T51, T52, K53, D78, T107, Q166, N185, D186, E189, S193, N207
		lowest energy	–163.4					

The DC1 configuration revealed an interaction interface involving PB1-F2 residues Q5, E14, I27, Q28, K29, S34, S35, T36, Q37, D40, L72, and R73, together with VDAC1 residues D132, I133, A134, Y153, E158, K161, R163, V164, T165, Q166, S167, D186, and T188. Interestingly, the VDAC1 residues identified in DC1 are primarily associated with β-strands 10–11, paralleling the interface observed in previous experimental and biophysical studies of the VDAC2-BAK complex. There, deep mutational scanning and cysteine cross-linking pinpointed the VDAC2 β10–11 loop as critical for BAK binding.^34,35^ This similarity suggests a potentially conserved apoptotic interaction mechanism. Moreover, principal component analysis of unbound-VDAC1 also indicated that the same amino acid residues undergo large-scale dynamics. The preliminary response of VDAC1 to the presence of PB1-F2 in all docked conformations was further analyzed by residual fluctuations in the VDAC structure (Figure 4C,D). Again, no substantial difference in VDAC’s architecture was observed except for DC1, which showed increased fluctuations in the N-terminal region already known to be essential for channel gating.^25,81^ Importantly, here PB1-F2 does not directly interact with the N-terminal helix of VDAC1 (Figure 4E). Rather, the binding interface of PB1-F2 in DC1 appears to induce allosteric effects that drive movement of the N-terminal domain, reflecting its intrinsic gating-related dynamics, without implying direct modulation of channel gating. Although several docked conformations exhibited favorable docking scores and interface areas (Table 2) with variable contacting residues (Figure S5), DC1 was selected for extended simulations based on its comparatively stable interface during MD simulations, its ability to induce pronounced N-terminal fluctuations in VDAC1, and the biological relevance of its β10–11 interaction region, which has previously been implicated in apoptotic protein recognition in VDAC family members.

### In-Depth Analysis of PB1-F2 and VDAC1 Interactions

For extensive conformational space sampling, the DC1 conformation was simulated for roughly 10 μs. A distance-based principal component analysis (dPCA) revealed interface regions that contribute most to the structural variability and identified nine distinct clusters (C1–C9) (Figure S6). Stable conformations, for instance, C3, C4, coexisted with highly dynamic ones like C8, C9, each occupying separate regions in the reduced-dimensional space. The persistence of C4 for over 1.5 μs underscores the potential biological relevance of specific interaction modes.

To obtain energetically favorable configurations, we plotted the free energy landscape of the simulated data derived from the radius of gyration (Rg) and the root-mean-square deviation (RMSD) of the PB1-F2/VDAC1 complex, which highlighted five distinct metastable states (MS1-MS5) (Figure 5A). These minima represent energetically favorable conformations sampled during the simulation. MS1, located at the global free energy minimum, corresponds to the most stable state where the NTD of PB1-F2 aligns parallel to the β-strands of VDAC1, targeting channel lumen forming multiple stabilizing interactions, including hydrogen bonds and electrostatic contacts with residues on the channel wall. MS2, MS3, and MS4 (Figure S7) represent intermediate states where PB1-F2 shifts to partial peripheral contacts with VDAC1, with reduced structural compactness and stability compared to MS1. MS5, located at a higher Rg and RMSD region, depicted a configuration where PB1-F2 dissociates partially from the lumen and interacts transiently with VDAC1 residues on the cytoplasmic side (Figure S7). The distinct structural motifs in each state illustrate the flexibility and dynamic nature of the PB1-F2/VDAC1 interaction.

**Figure 5:**
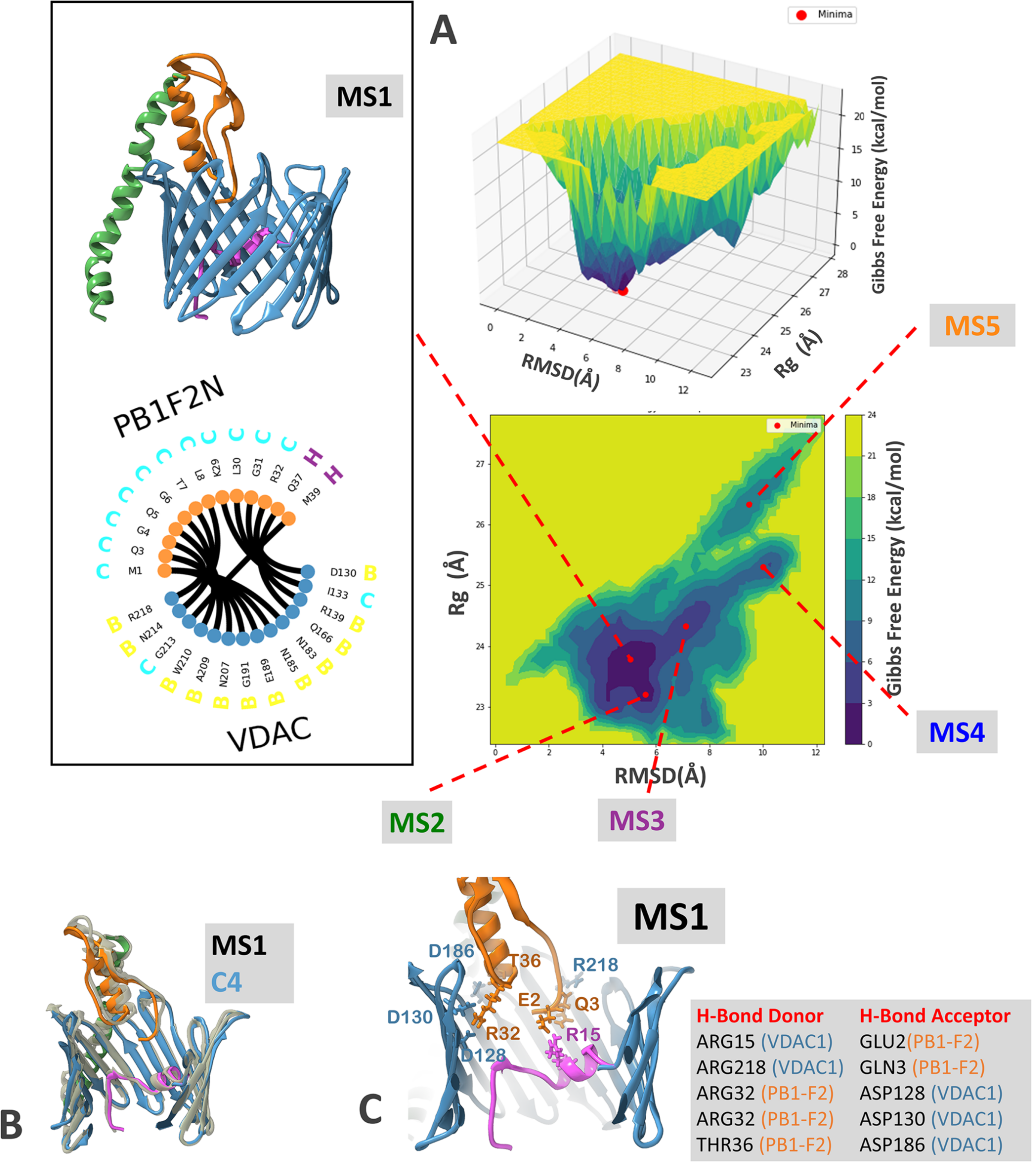
(A) Free Energy Landscape (FEL) derived from the radius of gyration (Rg) and the RMSD of DC1 sampled along 10.2 μs of MD data, along with the metastable states represented as **MS1-MS5** in the energy landscape. The energetically most favorable state, **MS1**, is highlighted in a box and supported by a wheel representation of contacting residues with their secondary structure (H: helix, C: Coil, and B: β Sheet), with a cartoon depiction of the PB1-F2/VDAC1 complex (color-coded based on spheres on wheel plot). (B) Overlay of C4 cluster central member on metastable state (MS1) on global free energy minimum. (C) Cartoon Depiction of PB1-F2 and VDAC1 interaction via hydrogen bonds in MS1 state supported by tabulated data for H-bond Bond donors and H-bond acceptors.

It is to be noted that MS1 aligns well with the cluster C4 from the dPCA (Figure 5B), with an estimated RMSD of 0.99 Å. Therefore, we further quantified the residence times (τ) of the MS1 state from the cluster assignment trajectory. MS1 was visited 368 times across the combined replicas. While the median lifetime is short, ∼0.2 ns, indicating frequent transitions, the distribution exhibits a pronounced long tail, with events persisting up to ∼670 ns with mean τ = 3.8 ns. These results suggest that MS1 represents a recurrent conformational substate within the dynamic ensemble that can intermittently achieve kinetic stabilization, despite its moderate, ∼12% population. The repeated sampling of MS1 across independent replicas and its overlap with the dPCA-derived C4 cluster support the stability of the dominant metastable ensemble, with no substantial conformational drift observed during the later stages of the simulations. In this configuration, PB1-F2 residues Arg32, Thr36, Glu2, and Gln3 form hydrogen bonds with VDAC1 residues Asp128, Asp130, Asp186, Arg218, and Arg15 (Figure 5C), anchoring PB1-F2 near the channel opening and potentially promoting conformational rearrangements that modulate VDAC1 dynamics. We also analyzed the correlated motions within VDAC1 by using linear mutual information correlation matrices.^82^ As a reference, the PB1-F2 unbound form of VDAC1 showed a minimal correlation between the residues, based on the 2.2 μs simulation data. Whereas, on the other hand, PB1-F2 bound VDAC1 in DC1 showed significant correlations between the amino acid residues (Figure 6A,B), these correlated motions were more profound between the N-terminal domain and the β-barrel residues that provide attachment to this domain, i.e., residues from 130 to 283. This exactly depicts the one-half of the β-barrel structure intersecting from the point of attachment of PB1-F2, thereby validating the functionally relevant interaction site for PB1-F2. This behavior was further validated by the root-mean-square fluctuation (RMSF) analysis for both PB1-F2 and VDAC1 amino acid residues before and after complex formation (Figure S8A,B), which reveals an increased flexibility of residues 1 to 140 of VDAC1 in the DC1 complex, consistent with the correlation matrices, which identified correlative motions within the pore architecture, focusing on amino acid residues ∼130 to 283. Notably, previous studies have proposed that the N-terminal α-helix controls transitions into elliptic β-barrel conformations that form the structural basis of the closed state of VDAC.^20,75^ Therefore, the enhanced N-terminal flexibility and correlated motions observed in the PB1-F2-bound state are consistent with gating-associated dynamical changes.

**Figure 6:**
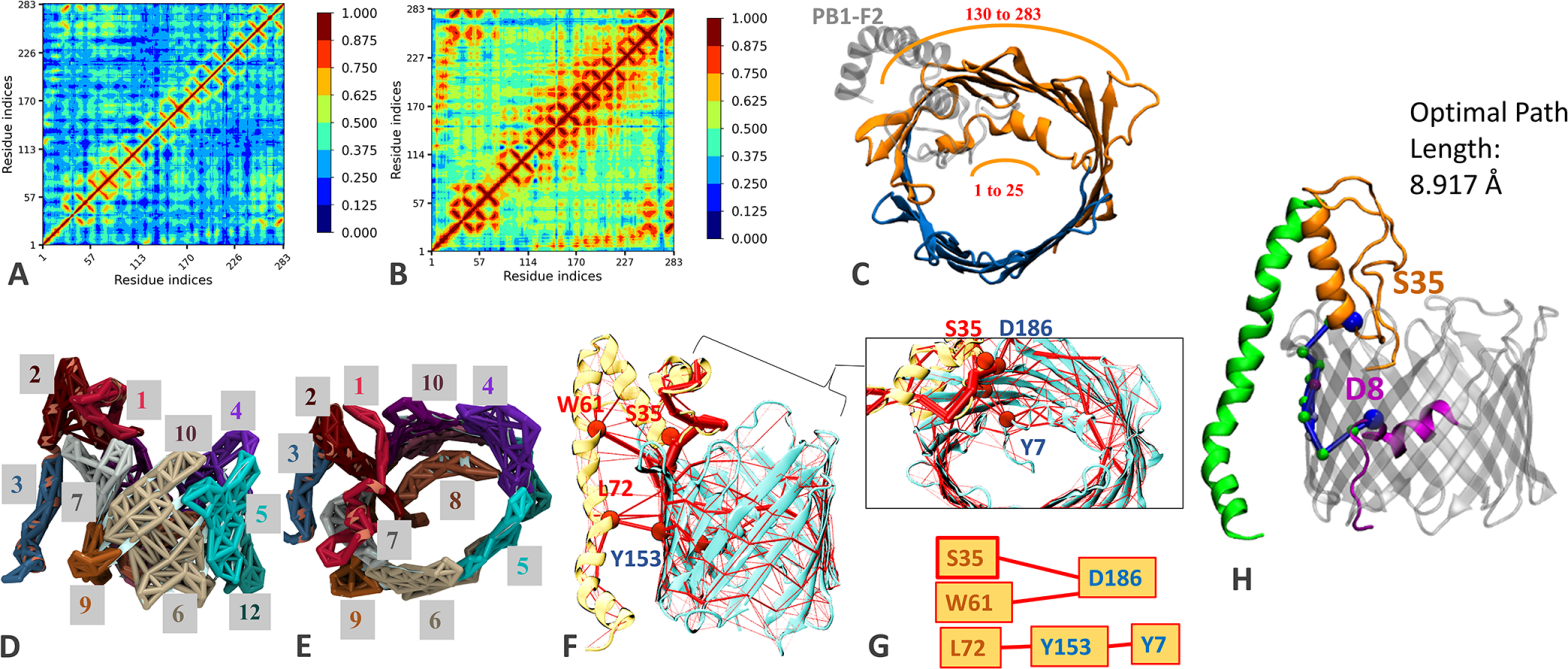
(A) Linear Mutual Information correlation matrix between Cα atoms of the unbound VDAC1 based on the 2.2 μs simulation data and (B) the VDAC1 bound to PB1-F2 based on the 10.2 μs simulation data (DC1), with (C) visual depiction of the residues involved in correlative motions in orange ribbons. (D, E) Cartoon representations of the correlation-based communities of PB1-F2-bound VDAC1, represented by different colors of the contacting edges in the dynamical network. (F) Depictions of the edge betweenness in which the edges are represented as red tubes with thick tubes bearing high betweenness values, along with a zoomed-in view of the VDAC1 focused on the N-terminal end. (G) Nodes with the highest betweenness values are highlighted, in which S35, W61, and L72 of PB1-F2 exhibited the highest betweenness values, respectively, connecting D186, Y153, and Y7 of VDAC1. (H) Visual depiction of optimal interaction path between S35 of PB1-F2 and D8 of VDAC1 computed from D12 μs MD data of DC1.

The PB1-F2/VDAC1 complex in DC1 was further evaluated via a dynamical network analysis to identify the essential residues and information pathways that mediate possible allosteric communication within the complex. The residue–residue correlation data are represented as a network with the C-α atoms at the nodes and the correlation as weighted edges. Analysis of the interaction network revealed communities composed of residues that are internally strongly correlated (Figure 6D,E). The first three communities belong to PB1-F2, and communities 4 to 12 belong to VDAC1 (Table 3). Notably, community no. 8 is associated with the VDAC1 N-terminal domain, closely connected to the residues of community no. 9 at the bottom of the β-barrel architecture facing the CTD (third community) of PB1-F2. Investigation of betweenness centrality provides evidence of the importance of different amino acid residues in mediating communication between different regions of the complex. In the PB1-F2 and VDAC1 complex, edges with the highest betweenness in the interface are the ones connecting S35, W61, L72 of PB1-F2 with Y153, D186, Y7 of VDAC1 (Figure 6F,G). S35 and W61 were connected to D186, whereas L72 was connected to Y153; this connection was further extended to Y7. These connections define a central communication hub and suggest probable long-range allosteric interactions within the complex. Figure 6H illustrates the optimal communication pathway between the PB1-F2 interaction site at S35 and the highly mobile N-terminal residue D8 (Figure S8B) of VDAC1, with a calculated path length of 8.92 Å. This pathway highlights a potential allosteric connection within the complex, whereas the possibility of PB1-F2 binding influencing the conformational states of VDAC1 through long-range interactions remains. Therefore, alternative pathways may exist, as suggested by the analysis of the betweenness centrality, further emphasizing the role of key residues in facilitating the communication between PB1-F2 and VDAC1.

**Table 3:** Community IDs and Their Corresponding Residue Ranges for PB1-F2 and VDAC1

community ID	residue ranges(s)
**1**	**PB1-F2:** 1–30
**2**	**PB1-F2:** 31- 64
**3**	**PB1-F2:** 65- 90
**4**	**VDAC1:** 23–26 221–245 260–277
**5**	**VDAC1:** 27–33 41–58 75–81 281–283
**6**	**VDAC1:** 34–40 59–74 82–86 97–99 100–115
**7**	**VDAC1:** 127–140 153–168
**8**	**VDAC1:** 1–22
**9**	**VDAC1:** 169–192 206–220
**10**	**VDAC1:** 172–181 193–205
**11**	**VDAC1:** 224–233 246–259 278–280
**12**	**VDAC1:** 87–96 116–126 141–152

### Pore Dynamics and Structural Deformity of VDAC1

Next, we investigated whether the PB1-F2-induced dynamics associated with the N-terminal helix of VDAC1 could affect its transport properties. To this end, we calculated the pore radius along MD trajectories for two systems: VDAC1 not bound to PB1-F2 (2.2 μs) and the selected docked conformation (DC1, 10.2 μs). Intriguingly, the DC1 complex exhibited subtle pore-size variability, particularly around the constriction zone formed by residues D8, A9, R15, and K20 (Figure 7A,B). To quantify this, the average minimum radius in the DC1 complex was determined to be 5.51 ± 0.69 Å, compared to 5.22 ± 0.51 Å in the unbound state. This difference is statistically significant (mean difference = 0.29 Å, 95% Confidence interval [0.27, 0.31], unpaired *t* test, t = −41.16, *p* < 0.0001), indicating an increased dynamic behavior upon PB1-F2 binding. The slightly enlarged average pore radius, together with the heightened fluctuation, suggests that PB1-F2 binding may influence the local pore architecture and potentially modulate VDAC1 gating and ion transport properties. Furthermore, we noted that deformations of the VDAC1 β-barrel were more pronounced in the DC1 simulation, as indicated by the calculated barrel ellipticity. A peak value of ∼0.29 was observed for DC1, significantly higher than the unbound state (∼0.20) (Figure 7C). These results align with previously reported data,^20,23^ which show that an increased β-barrel ellipticity is associated with partially collapsed or closed states of VDAC1, often linked to destabilization or subtle conformational changes in the N-terminal region involved in voltage gating.^23^

**Figure 7:**
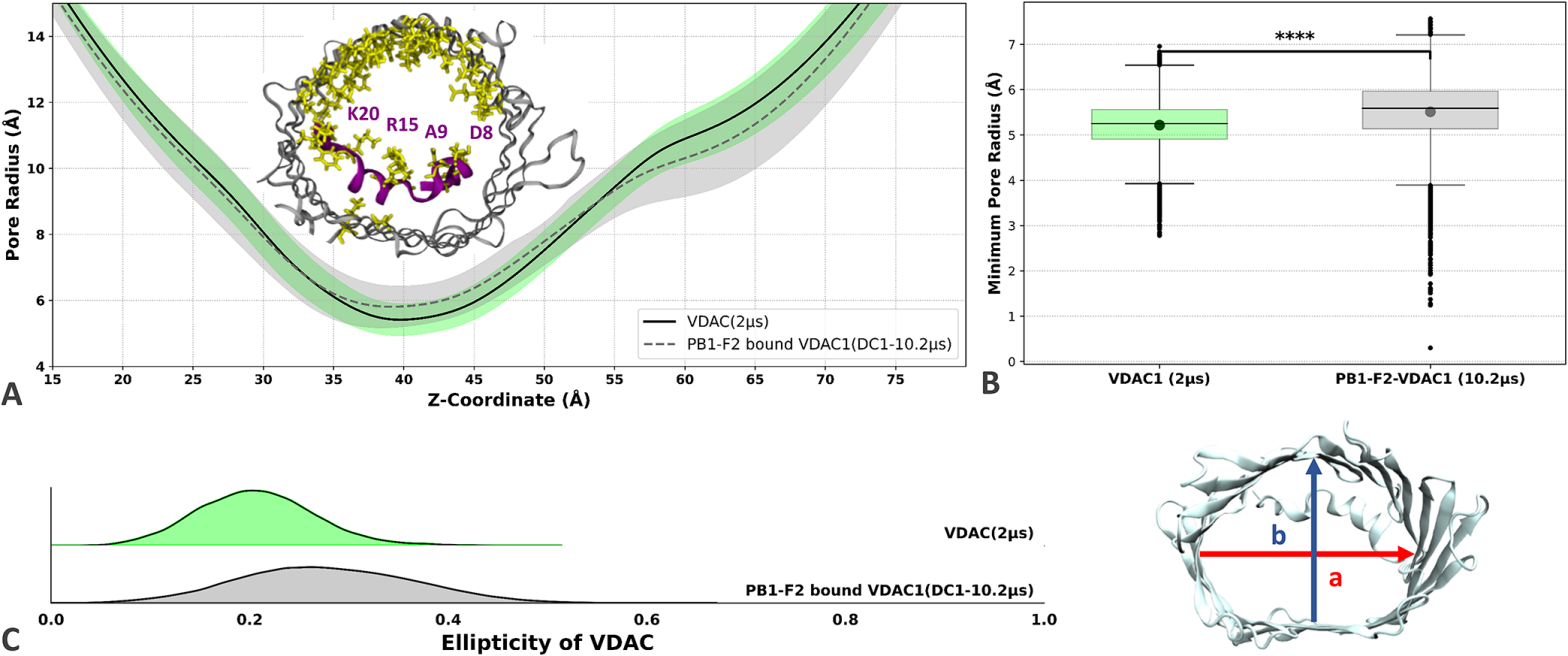
(A) Pore radius along the channel axis of VDAC1 in its PB1-F2 unbound state (green, 2 μs), along with PB1-F2 bound-state in DC1 complex (gray, 10.2 μs). The solid and dashed lines represent the mean pore radii, while the shaded areas indicate the standard deviations. VDAC1 is depicted in cartoon structure with pore-defining residues highlighted in yellow sticks, whereas the N-terminal domain is represented in purple ribbons and the rest of the channel in gray ribbons. (B) Minimum pore radius fluctuations at the constriction region of VDAC1 in the absence and presence of PB1-F2, where the boxes show the interquartile range, the horizontal lines indicate the median, and the whiskers the data spread. The circles mark the mean values, and statistical significance is shown (****: *p* < 0.0001, unpaired *t* test). (C) Distribution of VDAC1 ellipticity $\left(E=1-\frac{b}{a}\right)$ for the two systems, where conformations close to 0 represent a perfectly circular β-barrel structure, and those close to 1 represent an elliptic β-barrel structure.

### Electric Field as a Modulator of PB1-F2/VDAC1 Binding

Electrophysiology studies can provide critical insights into the transport properties and dynamics of channel proteins.^83^ Computational electrophysiology provides an atomistic view of the structural transitions underlying these dynamics and helps interpret the experimental data.^70,71^ VDAC1 is known to undergo gating transitions under applied external electric field,^46,48^ which mimics a potential physiological mitochondrial membrane potential, making it biologically relevant for studying voltage-dependent channel behavior. We first conducted applied field simulations on PB1-F2 unbound VDAC1, reproducing ionic conductivity and selectivity consistent with previous computational studies (Table 4). Experimental single-channel conductance values for VDAC1 range from 3.9 nS to 4.5 nS in a 1.0 M KCl solution.^84^ After validating our protocol, to investigate whether an applied voltage facilitates PB1-F2’s interaction with VDAC1, we performed simulations on the metastable state 1 (MS1) of DC1 complex under ± 100 mV and ± 75 mV, focusing on voltages where VDAC1 gating effects are more pronounced, i.e., at higher voltages.^84,85^ We analyzed the relative movement of the NTD of PB1-F2 with respect to the center of mass (COM) of VDAC1 by computing their distances over time.

**Table 4:** Comparison of Simulated Ionic Currents, Total Current, and Conductance of Only the VDAC1 System with Reported Literature Values,^61^ Along with Mean Conductance Computed at External Voltages of ± 100 mV and ± 75 mV for PB1F2-VDAC1 Complex (MS1 state)

voltage (mV)	*I*_K_ (nA)	reported *I*_K_ (nA)	*I*_Cl_ (nA)	reported *I*_Cl_ (nA)	*I*_total_ (nA)	reported *I*_total_ (nA)	conductance (nS)	reported conductance (nS)	calculated conductance for PB1F2-VDAC1 complex (MS1 state)
**–100**	–0.264	–0.132	0.282	0.366	0.545	0.498	5.45	4.98	4.67
**–75**	–0.177	–0.138	0.218	0.289	0.395	0.427	5.26	5.69	5.8
**–50**	–0.119	–0.056	0.143	0.203	0.261	0.259	5.22	5.18	-
**–25**	–0.05	0.013	0.077	–0.104	0.126	0.117	5.05	4.68	-
**+25**	0.051	0.053	–0.097	0.082	0.148	0.134	5.91	5.36	-
**+50**	0.152	0.088	–0.154	0.134	0.306	0.223	6.12	4.46	-
**+75**	0.159	0.126	–0.254	0.183	0.412	0.31	5.5	4.13	5.57
**+100**	0.184	0.166	–0.285	0.275	0.469	0.442	4.69	4.42	5.27

Our results indicate that MS1 displayed a stable interaction, with minimal fluctuations in distance (Figure 8A). Interestingly, under negative potentials, a reduction in distances of approximately 2–4 Å was observed, suggesting that negative voltages might promote a tighter association between PB1-F2 and VDAC1. These findings highlight the significance of the specific binding interface observed in MS1, which is stabilized by hydrogen bonds and electrostatic interactions. Especially, these interactions become more stable at external voltages, suggesting a voltage-dependent regulation of the PB1-F2/VDAC1 complex.

**Figure 8:**
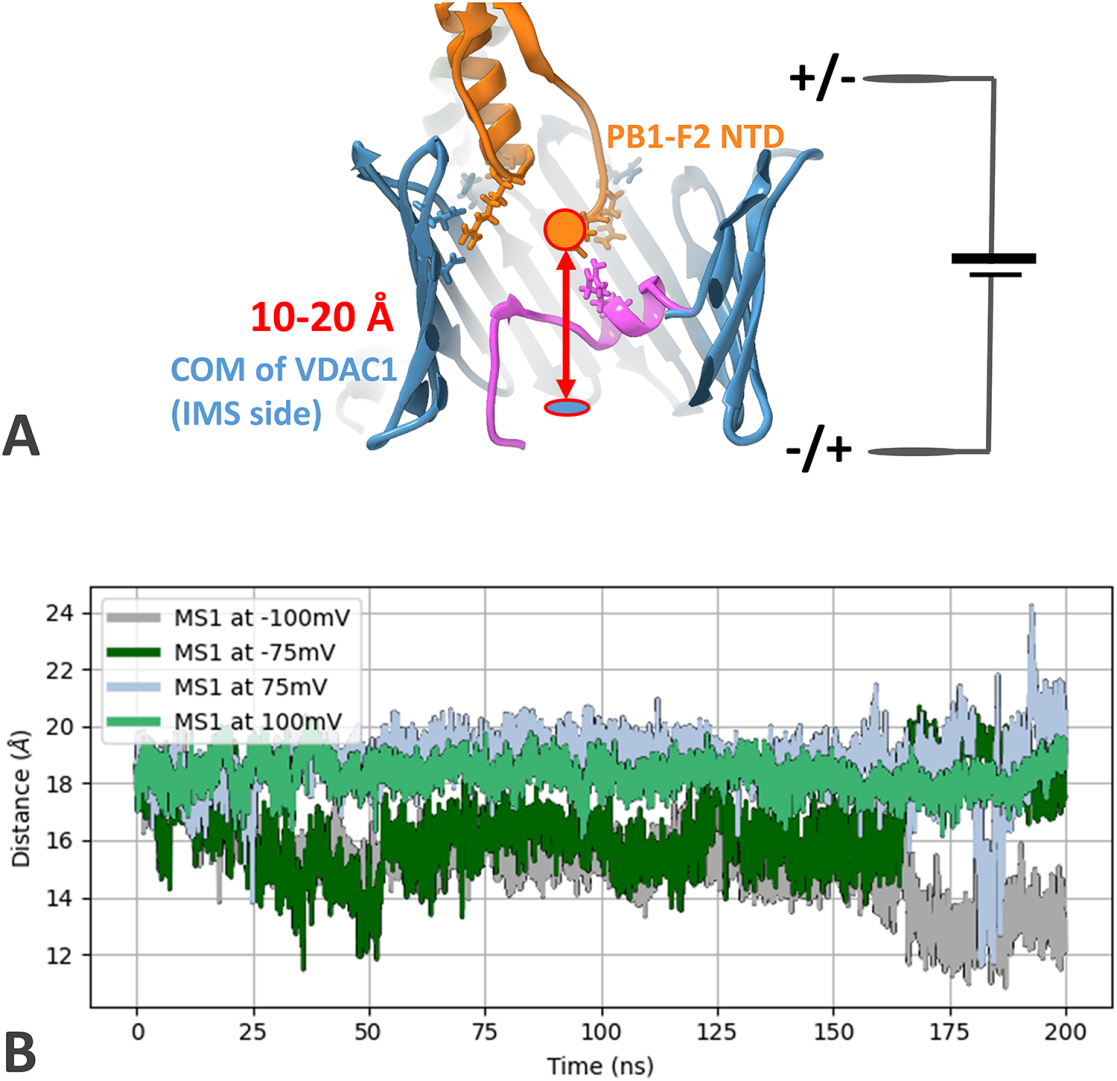
(A) Cartoon depiction of the distance between the COM of the IMS side of VDAC1 and the N-terminal end of PB1-F2 under an applied electric field. (B) Distance between the center of mass (COM) of VDAC1’s intermembrane space (IMS) side and the N-terminal end of PB1-F2 computed at external voltages of ± 100 mV and ± 75 mV for MS1 (metastable state 1) from the 10.2 μs simulation data of DC1.

Despite voltage-dependent stabilization of this complex, no substantial shift in ionic selectivity was observed compared to the unbound VDAC1, and conductance values remained within the same range. Thus, PB1-F2 binding appears to modulate structural and dynamic features of VDAC1 without markedly altering its ion selectivity under the simulated conditions. These findings suggest a voltage-sensitive stabilization of the complex that may subtly influence channel behavior, while larger functional consequences remain to be established experimentally.

Moreover, the lipid composition may further modulate these interactions. In particular, cardiolipin has been implicated in apoptotic signaling^91,50^ and VDAC dynamics,^90^ potentially affecting the stability or electrostatic environment of the PB1-F2/VDAC1 complex, especially under voltage-dependent conditions. Future studies using more realistic membrane models may therefore clarify lipid-mediated modulations of PB1-F2/VDAC1 interactions.

Although PB1-F2 binding does not induce a complete loss of VDAC1 conductance in our simulations, the observed gating-biased conformational ensemble may still have functional consequences at the systems level. VDAC1 is a central regulator of mitochondrial ion exchange and Ca^2+^ homeostasis,^31,79,86^ and subtle alterations in barrel ellipticity and N-terminal dynamics could influence ion selectivity or local electrostatic profiles, potentially biasing Ca^2+^ flux under physiological membrane potential. In addition to its channel function, VDAC1 serves as a key interaction hub for apoptotic regulators, including pro-apoptotic proteins such as BAX and BAK,^33,35,38,39^ and binds hexokinase at the outer mitochondrial membrane, thereby coupling metabolism to cell survival.^73,87,88^ Conformational priming of VDAC1 by PB1-F2 may therefore influence its interaction landscape, potentially modulating these regulatory associations. Under cellular stress conditions, such allosteric modulation could shift the equilibrium among VDAC1 regulatory states, thereby sensitizing mitochondria to apoptotic signaling without requiring complete channel closure.

## Conclusion

PB1-F2 is a pro-apoptotic protein encoded by the influenza A virus that has been implicated in mitochondrial dysfunction and immune regulation. One of its key interaction partners involved is VDAC1, which is a crucial outer mitochondrial membrane protein responsible for metabolite and ion exchange. Previous work has reported that PB1-F2 binds to VDAC1,^3^ suggesting a potential link between this interaction and altered mitochondrial membrane permeability and apoptosis induction through cytochrome c release. However, the structural and mechanistic details of this interaction remain unclear.

In this study, we investigated molecular interactions between PB1-F2 and VDAC1 using combinations of steered molecular dynamics simulations, protein–protein docking analysis, and unbiased and voltage-driven molecular dynamics simulations. Initial steered MD simulations enforced the approach of PB1-F2 toward the biologically relevant E73 amino acid residue of VDAC1 via its CTD, with electrostatic interactions governing the initial contact. Further unbiased MD simulations demonstrated, however, that PB1-F2 does not form a stable interaction with the E73 residue of VDAC1, despite previous reports suggesting its role as a key interaction site.^62,73,78^ Instead, a docking approach followed by MD simulations revealed an alternative binding mode, in which PB1-F2 primarily interacts with VDAC1 via its β-strands 10–11 and adjacent loop regions. At the same time, the membrane-targeting sequence (MTS) of PB1-F2 remains positioned in the membrane.

The selection of the most stable docking conformation (DC1) for long-term simulation provided deeper insight into the allosteric effects induced by PB1-F2 binding for a likely gating mechanism of VDAC1. A principal component analysis and dynamical network analysis identified key interaction hotspots, particularly residues on the β-barrel of VDAC1. The interaction of PB1-F2 with these regions resulted in significant conformational changes in the pore, characterized by a relative increase in channel ellipticity and changes in the constriction at the pore lining, indicative of a shift toward a closure-like conformational ensemble. Importantly, our findings suggest that PB1-F2 binding is associated with long-range structural effects rather than direct pore occlusion. Through free energy landscape analysis, we identified a metastable state (MS1) corresponding to the global free-energy minimum, populated for approximately 12% of the simulation time. This state is characterized by strengthened interactions between PB1-F2 and the N-terminal region of VDAC1 and represents a recurrent lumen-proximal binding configuration within the dynamic ensemble. Although individual MS1 visits are typically short-lived, intermittent stabilization events reaching hundreds of nanoseconds suggest that this configuration can persist sufficiently long to permit local structural rearrangements. While apoptotic signaling occurs on longer cellular time scales, such kinetically stabilized interactions at the molecular level may contribute to cumulative modulation of VDAC1 conformational dynamics.

Furthermore, applied-field simulations revealed that PB1-F2 binding is voltage-sensitive. Particularly in the MS1 configuration, negative potentials enhance the association between PB1-F2 and VDAC1 by stabilizing electrostatic interactions. This finding aligns with the physiological relevance of membrane potential fluctuations during apoptosis,^89^ further supporting the role of PB1-F2 in mitochondrial dysfunction. However, its effect on ionic conductance was minimal. Unlike α-synuclein and tubulin, which have been reported to partially occlude the VDAC1 pore and alter its conductance and selectivity,^28–30^ the positively charged PB1-F2 did not disrupt ionic selectivity when interacting within the pore lumen. Collectively, the correlation between the PB1-F2 binding and the VDAC1 pore dynamics suggests that the interaction may influence channel conductance properties. However, the broader consequences for mitochondrial permeability or apoptosis cannot be inferred from the present simulations.

By elucidating the structural and dynamic basis of the PB1-F2 and VDAC1 interaction, we contribute to a broader understanding of how influenza A viruses exploit host apoptotic pathways. A schematic representation of the proposed mechanistic cascade, from PB1-F2 binding to VDAC1 conformational modulation, subsequent perturbation of mitochondrial permeability, and the potential release of apoptogenic factors such as cytochrome c, is presented in Figure S9. Given that experimental evidence for a direct PB1-F2/VDAC1 interaction remains limited, future studies should aim to validate these findings through independent biochemical and cellular approaches and to further explore the role of PB1-F2 in modulating VDAC1 function under varying cellular conditions. A deeper understanding of this interaction may provide new insights into viral pathogenesis and potential therapeutic strategies.

## Supplementary Material





## Notes

(A) Principal Component Analysis of PB1-F2, sorted molecular movements along the eigenvectors corresponding to the largest variance in conformational space; (B–D) projections of the MD data on the first 3 principal components, where the color gradient between blue, white, and red represents the scanning of conformations in chronological order. (E) Principal Component Analysis of VDAC1, sorted molecular movements along the eigenvectors corresponding to the largest variance in conformational space. (F–H) Same as (B–D) but for VDAC1 (Figure S1); (A) Short-range electrostatic energy between PB1-F2 and VDAC1, (B) hydrogen bond autocorrelation function, and (C) number of hydrogen bonds over the course of the 2 μs simulation following SMD1 (Figure S2); (A, D, G, J, M) distances between the NTD of PB1-F2 and the COM of IMS side of VDAC1 over 200 ns simulations. (B, E, H, K, N) Structural snapshots depicting interaction sites and spatial arrangements of PB1-F2 (colored helices based on snapshots 1–5) with VDAC1 (gray). The residue E73 is highlighted as red spheres, while the PB1-F2 chains are shown in distinct colors to differentiate the snapshots 1–5. (C, F, I, L, O) Residue contact maps illustrating key interacting residues between PB1-F2 and VDAC1 during the simulations. Each row represents horizontally an aligned analysis and visualizations corresponding to a specific simulation window (Figure S3); (A–E) Short-range electrostatic interaction energy profiles from snapshots 1–5 (Figure S4); (A–D) Cartoon representations of the PB1-F2 and VDAC1 complexes from DC2–5, along with their wheel representations of contacting residues with their secondary structure (H: helix, C: coil, and B: β sheet), with contacting residues represented as spheres (color-coded based on protein domains) (Figure S5); distance-based PCA, where all clusters from C1 to C9 are projected on the first five principal components from dPC1 to dPC5, and the clusters are represented in cartoon structures, color-coded according to cluster IDs (Figure S6); cartoon representations of the PB1-F2/VDAC1 complexes corresponding to metastable states MS2 to MS5 identified from the free energy landscape (FEL) constructed using the radius of gyration (Rg) and RMSD as reaction coordinates. Further supported by their wheel representations of contacting residues, annotated according to their secondary structural elements (H: helix; C: coil; B: β-sheet) (Figure S7); (A) Residual fluctuations of PB1-F2 in its VDAC1 unbound state as well as in the VDAC1-bound state in DC1 complex. (B) Residual fluctuations of VDAC1 in its PB1-F2 unbound state, along with the PB1-F2 bound state in DC1 complex (Figure S8); a schematic illustration summarizing the proposed cascade linking PB1-F2 binding to mitochondrial apoptotic signaling (Figure S9); the viral protein PB1-F2 associates with VDAC1 at the mitochondrial outer membrane (OMM), interacting near the β-barrel region (Stage 1); PB1-F2 binding induces allosteric modulation of VDAC1, leading to increased N-terminal mobility and deformation of the β-barrel from a circular to a more elliptical conformation (Stage 2); these conformational changes are proposed to perturb mitochondrial outer membrane permeability and influence membrane potential (ΔΨm) (Stage 3); the resulting mitochondrial dysfunction may facilitate the release of Cytochrome c into the cytosol, promoting apoptosome formation and activation of the apoptotic signaling cascade. The figure summarizes the hypothesized structural-functional pathway linking PB1-F2 binding to mitochondrial apoptosis induction (Stage 4) (PDF)
DC1 transitioning from MS1 to MS5 (GIF)
