# Supplementary material for: Computational Insights into PB1-F2 Mediated Modulation of VDAC1: An Allosteric Mechanism in Mitochondrial Dysfunction
Source: ACS Omega. 2026 Jun 30;11(27):40694–710. doi: 10.1021/acsomega.6c04195 (PMC13382746; doi:10.1021/acsomega.6c04195)
Supplement: Supplementary file 1 [file ao6c04195_si_001.pdf]

Supplementary Information

Computational Insights into PB1-F2 Mediated Modulation of VDAC1: An Allosteric Mechanism in Mitochondrial Dysfunction

Sehrish Jamal [a, b], Syed Tarique Moin [b], and Ulrich Kleinekathöfer [a*]

[a] School of Science, Constructor University, 28759 Bremen, Germany

[b] Third World Center for Science and Technology, H.E.J. Research Institute of Chemistry International Center for Chemical and Biological Sciences, University of Karachi, Karachi 75270, Pakistan

* Corresponding author: Ulrich Kleinekathöfer

Email: ukleinekathoefer@constructor.university

ORCID: Ulrich Kleinekathöfer: 0000-0002-6114-7431

Supplementary Analysis of NTD Pulling Simulations (SMD2)

To determine whether the NTD of PB1-F2 remains inside the VDAC1 lumen and potentially influences channel gating, we computed the distances between the PB1-F2 NTD and the center of mass of the intermembrane space (IMS) side of VDAC1 (Figure S3). The results revealed fluctuations in these distances over time, indicating that PB1-F2 engages in dynamic interactions with VDAC1. While some trajectories showed early stabilization of the NTD (e.g., Figure S3-D, G, M), others may require longer timescales to fully capture potential stable binding, as also reflected in the short-range electrostatic interaction energy profiles (Figure S4). Thus, these findings indicate variability in the interaction behavior and highlight the need for extended simulations to determine the persistence of biologically relevant contacts.

Residue contact maps (Figure S3) reveal that specific PB1-F2 residues, such as R32, P33, S34, and L38, frequently interact with N79, P105, and N106 of VDAC1. The primary interactions occur with residues on the β5 strand and the β7 loop of VDAC1, which remain consistent following SMD1, suggesting these regions provide structural support for the PB1-F2 attachment. The observed interactions and distance fluctuations indicate that the NTD of PB1-F2 may not form sustained contacts within the VDAC1 lumen. Although these findings do not exclude a potential involvement of PB1-F2 NTD in channel modulation, the current analysis provides no evidence for stable interactions with the N-terminal α-helix of VDAC1 (region essential for channel gating) when PB1-F2 NTD approaches from the cytosolic side with its CTD oriented toward the membrane-proximal E73 residue.

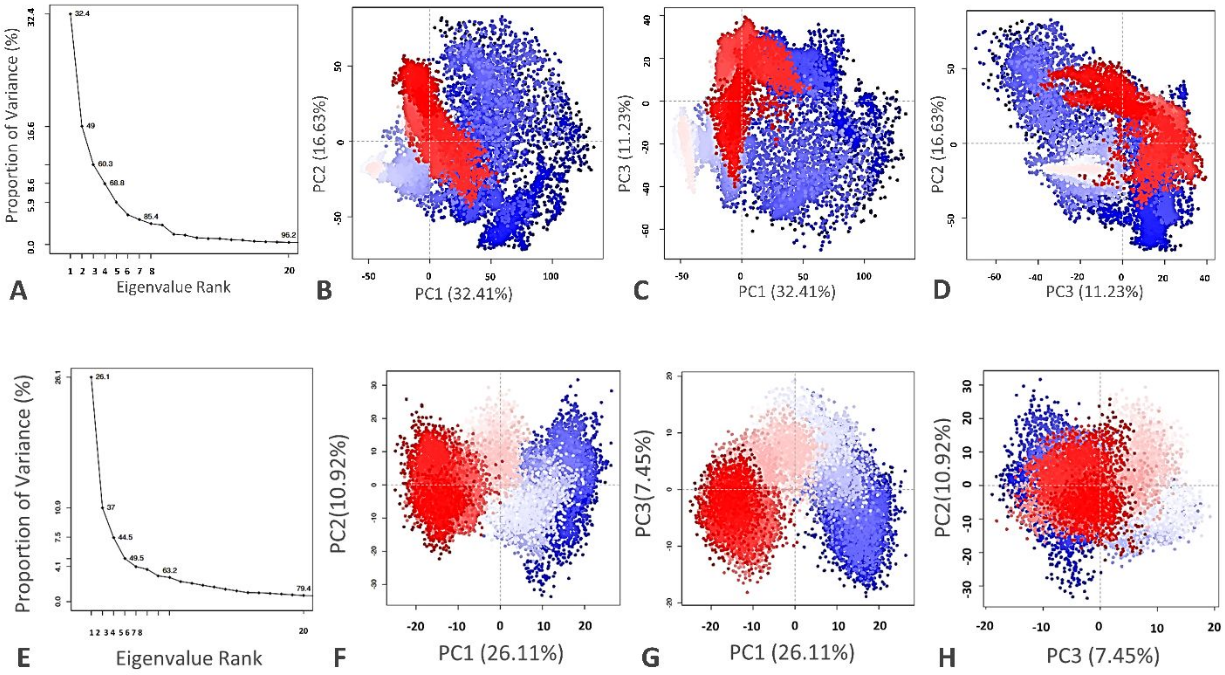

Figure S1: ***(A)*** *Principal Component Analysis of PB1-F2, sorted molecular movements along the eigenvectors corresponding to the largest variance in conformational space.* ***(B-D)*** *projections of the MD data on the first 3 principal components, where the color gradient between blue, white, and red represents the scanning of conformations in chronological order.* ***(E)*** *Principal Component Analysis of VDAC1, sorted molecular movements along the eigenvectors corresponding to the largest variance in conformational space.* ***(F-H)*** *Same as* ***(B-D)*** *but for VDAC1.*

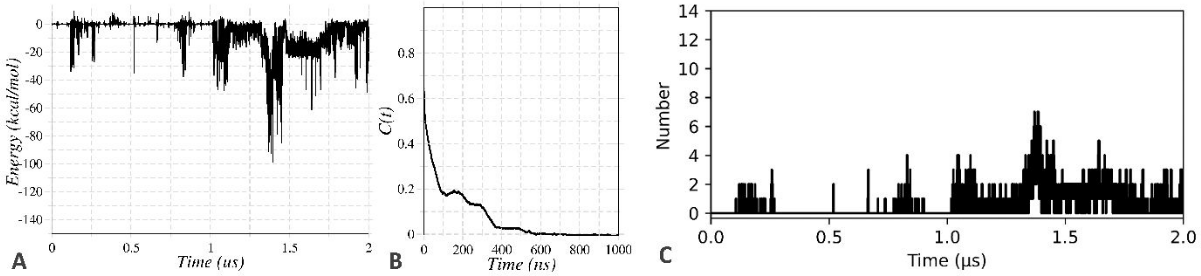

Figure S2: ***(A)*** *Short-range electrostatic energy between PB1-F2 and VDAC1,* ***(B)*** *hydrogen bond autocorrelation function, and* ***(C)*** *number of hydrogen bonds over the course of the 2 μs simulation following SMD1.*

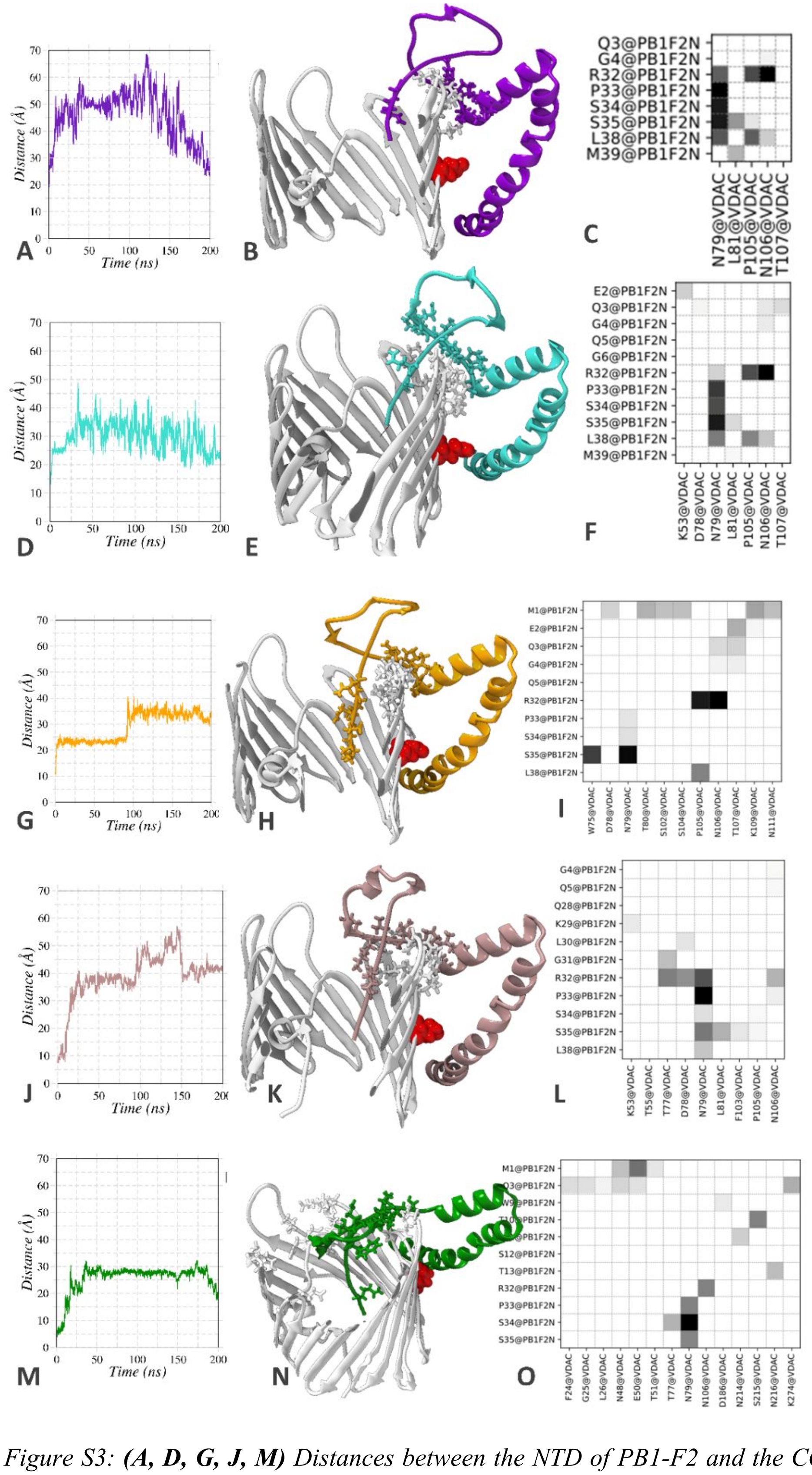

Figure S3: ***(A, D, G, J, M)*** *Distances between the NTD of PB1-F2 and the COM of IMS side of VDAC1 over 200 ns simulations.* ***(B, E, H, K, N)*** *Structural snapshots depicting interaction sites and spatial*

arrangements of PB1-F2 (colored helices based on snapshots 1-5) with VDAC1 (gray). The residue E73 is highlighted as red spheres, while the PB1-F2 chains are shown in distinct colors to differentiate the snapshots 1-5. ***(C, F, I, L, O)*** *Residue contact maps illustrating key interacting residues between PB1-F2 and VDAC1 during the simulations. Each row represents horizontally an aligned analysis and visualizations corresponding to a specific simulation window.*

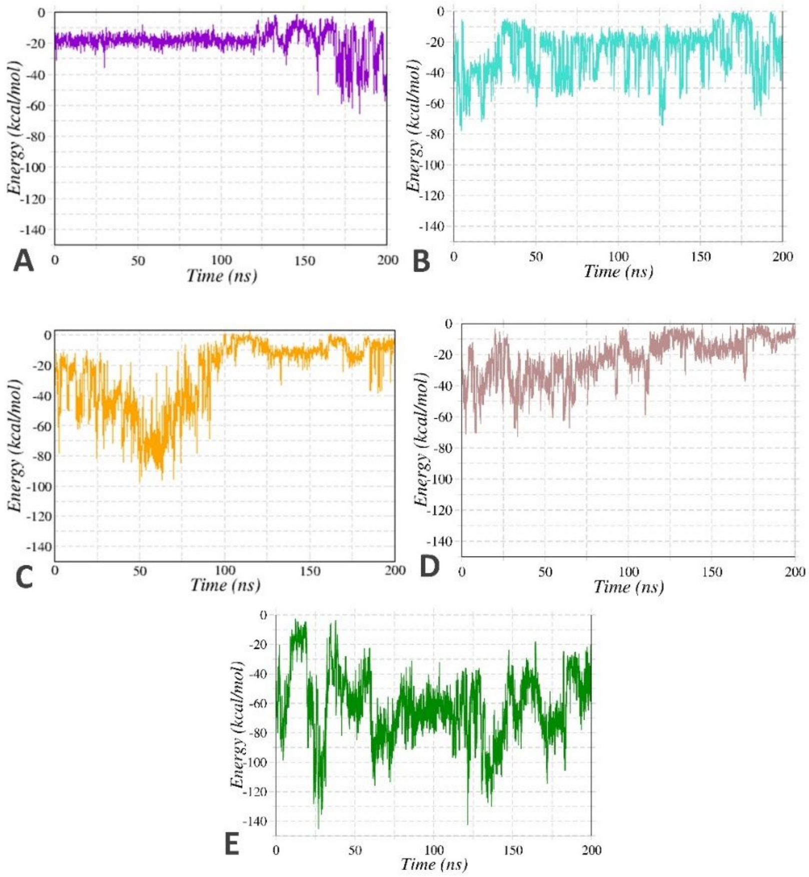

Figure S4: ***(A, B, C, D, E)*** *Short-range electrostatic interaction energy profiles from snapshots 1-5.*

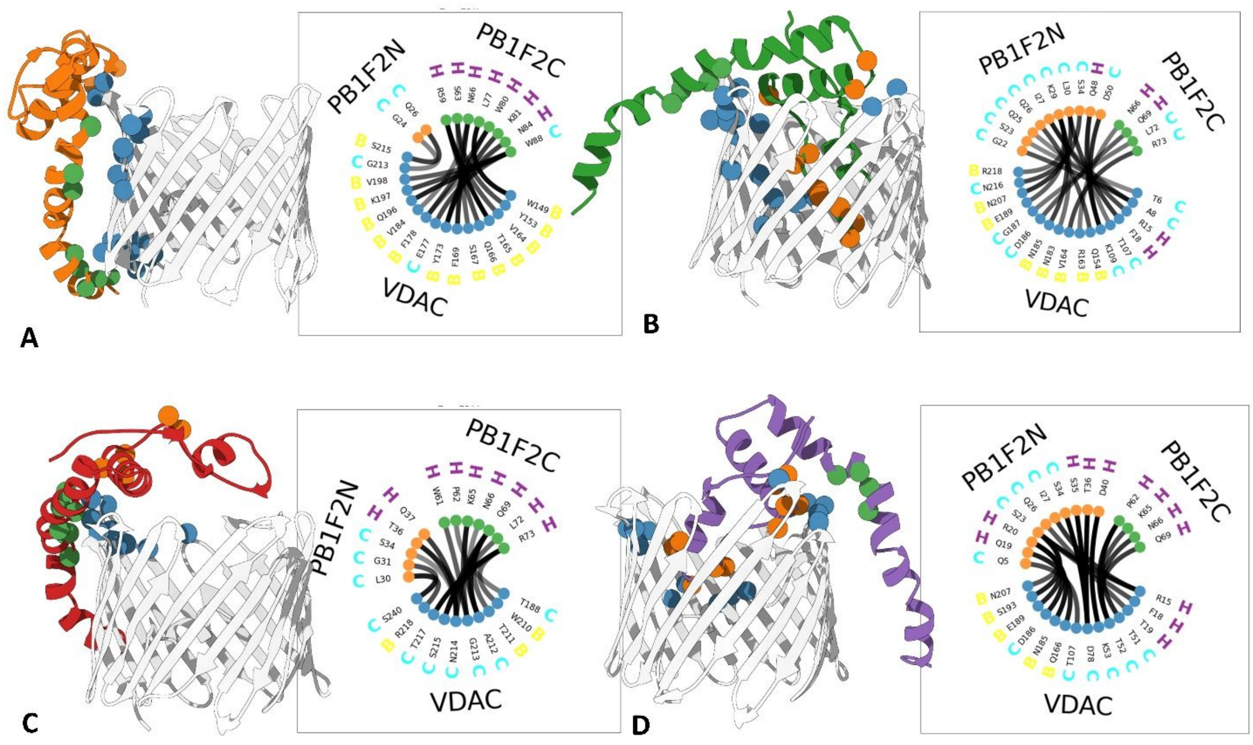

Figure S5: ***(A-D)*** *Cartoon representations of the PB1-F2 and VDAC1 complexes from DC2-5, along with their wheel representations of contacting residues with their secondary structure (H: helix, C: coil, and B: beta sheet), with contacting residues represented as spheres (color-coded based on protein domains).*

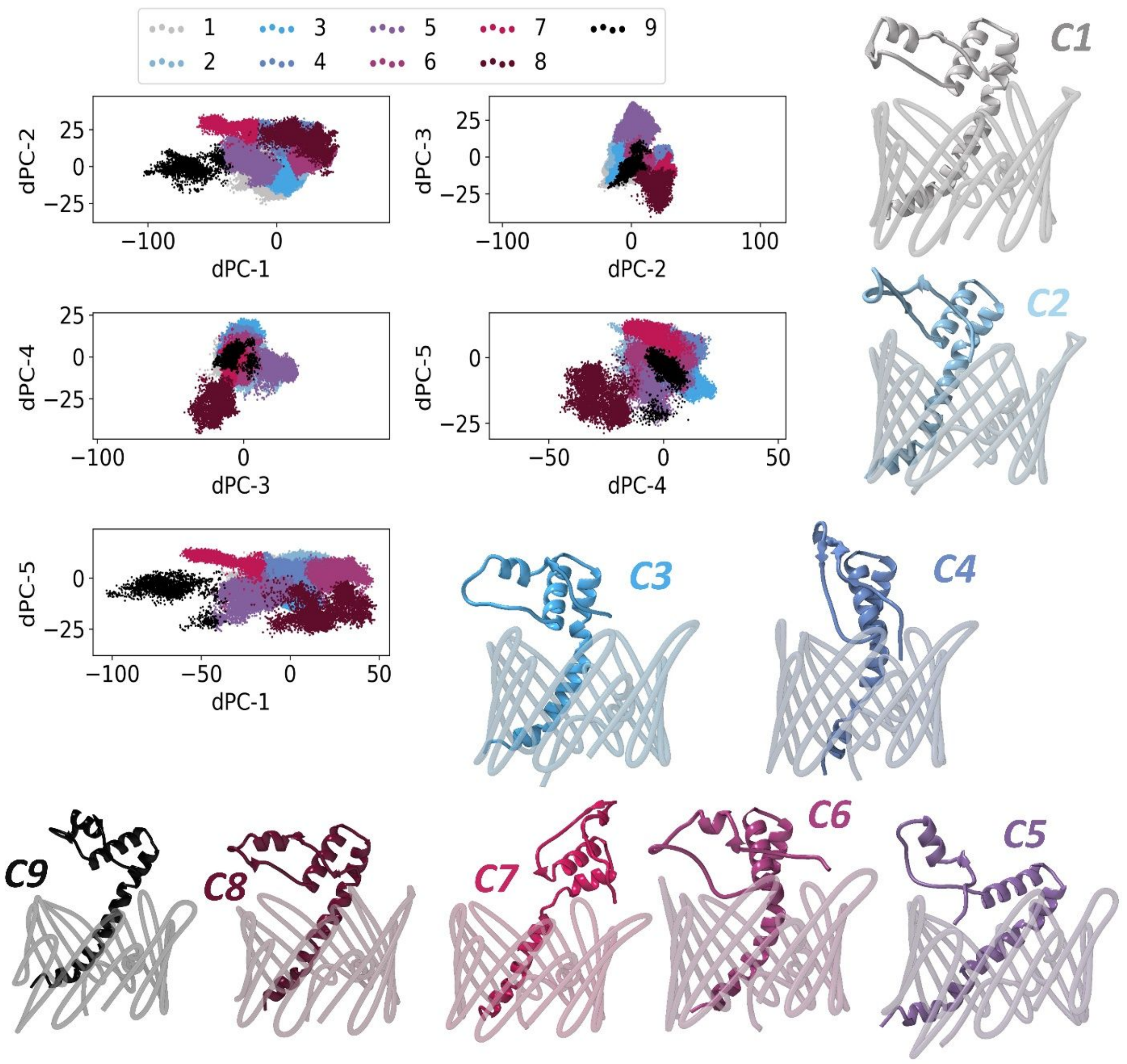

Figure S6: Distance-based PCA, where all clusters from C1 to C9 are projected on the first five principal components from dPC1 to dPC5, and the ***c****lusters are represented in cartoon structures, color-coded according to cluster IDs.*

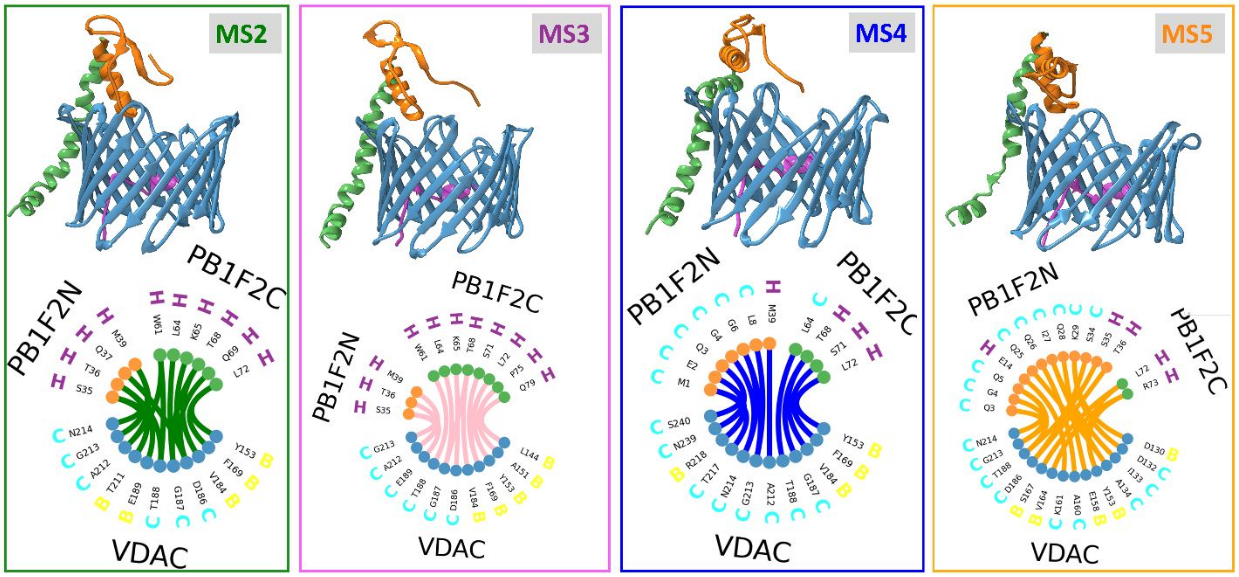

Figure S7: Cartoon representations of the PB1-F2/VDAC1 complexes corresponding to metastable states ***MS2*** *to* ***MS5*** *identified from the free energy landscape (FEL) constructed using the radius of gyration (Rg) and RMSD as reaction coordinates. Further supported by their wheel representations of contacting residues, annotated according to their secondary structural elements (H: helix; C: coil; B: β-sheet).*

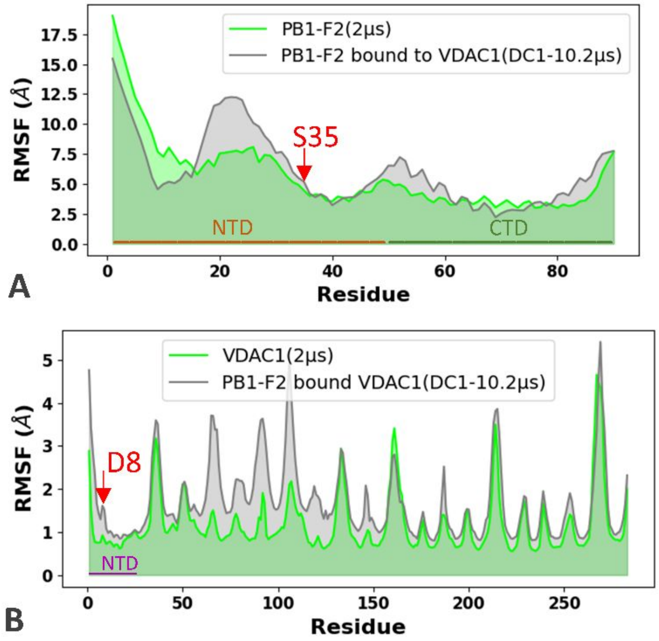

Figure S8: ***(A)*** *Residual fluctuations of PB1-F2 in its VDAC1 unbound state as well as in the VDAC1-bound state in DC1 complex.* ***(B)*** *Residual fluctuations of VDAC1 in its PB1-F2 unbound state, along with the PB1-F2 bound state in the DC1 complex.*

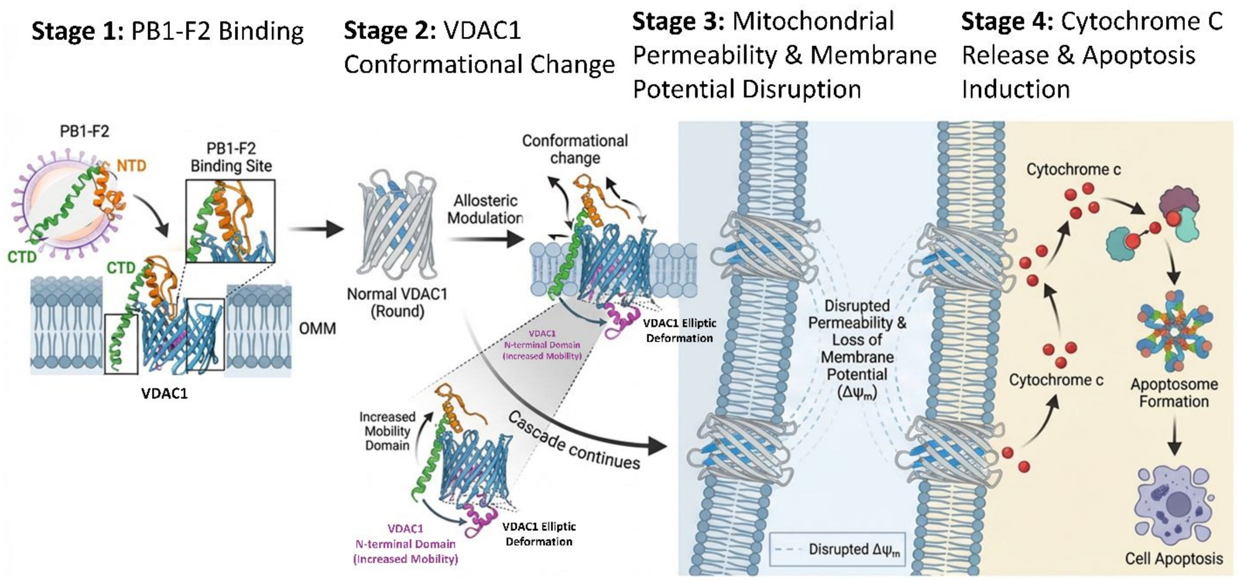

Figure S9: A schematic illustration summarizing the proposed cascade linking PB1-F2 binding to mitochondrial apoptotic signaling. ***Stage 1****: The viral protein PB1-F2 associates with VDAC1 at the mitochondrial outer membrane (OMM), interacting near the β-barrel region.* ***Stage 2****: PB1-F2 binding induces allosteric modulation of VDAC1, leading to increased N-terminal mobility and deformation of the β-barrel from a circular to a more elliptical conformation.* ***Stage 3****: These conformational changes are proposed to perturb mitochondrial outer membrane permeability and influence membrane potential (ΔΨm).* ***Stage 4****: The resulting mitochondrial dysfunction may facilitate the release of Cytochrome c into the cytosol, promoting apoptosome formation and activation of the apoptotic signaling cascade. The figure summarizes the hypothesized structural-functional pathway linking PB1-F2 binding to mitochondrial apoptosis induction.*
