# Supplementary figures and images for: Computational Insights into PB1-F2 Mediated Modulation of VDAC1: An Allosteric Mechanism in Mitochondrial Dysfunction
Source: ACS Omega. 2026 Jun 30;11(27):40694–710. doi: 10.1021/acsomega.6c04195 (PMC13382746; doi:10.1021/acsomega.6c04195)

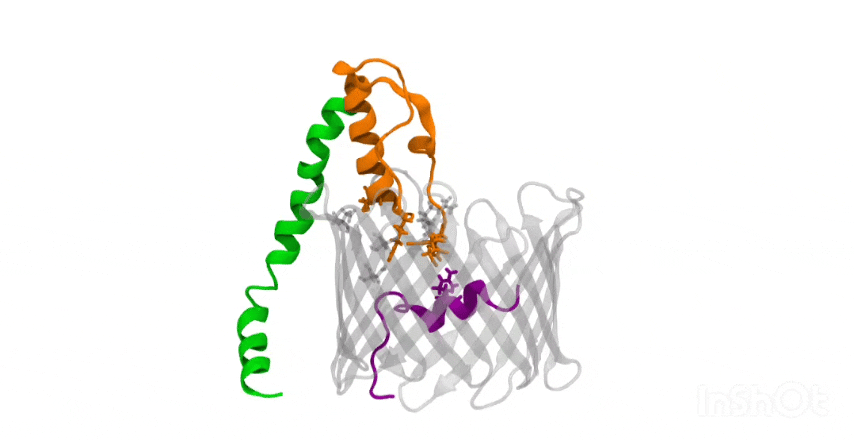

Supplement: Supplementary file 2 [file ao6c04195_si_002.gif]
